# Quantitative proteomic changes in LPS-activated monocyte-derived dendritic cells: A SWATH-MS study

**DOI:** 10.1038/s41598-019-40773-6

**Published:** 2019-03-13

**Authors:** Swati Arya, Dagmara Wiatrek-Moumoulidis, Silvia A. Synowsky, Sally L. Shirran, Catherine H. Botting, Simon J. Powis, Alan J. Stewart

**Affiliations:** 1 School of Medicine, University of St Andrews, St Andrews, KY16 9TF UK; 2 Biomedical Sciences Research Complex, University of St Andrews, St Andrews, KY16 9ST UK

## Abstract

Dendritic cells are key immune cells that respond to pathogens and co-ordinate many innate and adaptive immune responses. Quantitative mass spectrometry using Sequential Window Acquisition of all THeoretical fragment-ion spectra-Mass Spectrometry (SWATH-MS) was performed here to determine the global alterations in monocyte-derived dendritic cells (moDCs) in response to stimulation with lipopolysaccharide (LPS). A moDC library of 4,666 proteins was generated and proteins were quantified at 0, 6 and 24 h post-LPS stimulation using SWATH-MS. At 6 h and 24 h post-LPS exposure, the relative abundance of 227 and 282 proteins was statistically significantly altered (p-value ≤ 0.05), respectively. Functional annotation of proteins exhibiting significant changes in expression between the various time points led to the identification of clusters of proteins implicated in distinct cellular processes including interferon and interleukin signalling, endocytosis, the ER-phagosome pathway and antigen-presentation. In SWATH-MS major histocompatibility complex (MHC) class I proteins were highly upregulated at 24 h, whilst MHC class II proteins exhibited comparatively fewer changes over this period. This study provides new detailed insight into the global proteomic changes that occur in moDCs during antigen processing and presentation and further demonstrates the potential of SWATH-MS for the quantitative study of proteins involved in cellular processes.

## Introduction

Tissue-resident immature dendritic cells (DCs) exhibit a very high capacity to capture exogenous and cellular antigens through endocytosis and phagocytosis upon engagement of surface receptors. Antigens are recognized through pattern recognition receptors including the toll like receptor (TLR) family^1^. Immature DCs are highly phagocytic, however their antigen presenting ability is very limited. After antigen recognition, immature DCs begin a maturation process which can be divided into five phases^2^. Firstly, the morphology of DCs changes whereby the cells grow and develop cytoplasmic projections, a process involving cytoskeleton rearrangement. In this first phase cell motility increases by the loss of adhesive molecules^3^. In the second phase, maturing DCs express T-cell co-stimulatory molecules on the cell surface^4^. The third phase is characterized by migration to the lymph nodes and spleen, which enables cells to enter lymphatic vessels^5^. In the fourth phase, DCs express major histocompatibility complex (MHC) class II antigen presenting molecules on their cell surface and in the final phase chemokines and cytokines are secreted^4^. At this point, DCs become fully mature and are limited in their ability to take up new antigens but are ready to present the processed antigens to chemo-attracted, antigen-specific T-cells to therefore initiate the immune response^6^. Overall DCs are considered as mature when they can activate T-cells through distinct mechanisms.

To provide insight into the cellular mechanisms driving DC maturation a number of studies have been carried out examining proteomic changes that occur in DCs during this process. Several of these studies have utilized electrophoresis-based protein separation techniques, such as 2D-gel electrophoresis coupled with protein identification using mass spectrometry-based approaches^7–10^. More recently, approaches such as MudPIT (multi-dimensional protein identification technology) have been used^4^. These DC proteomic studies have focused on whole cell lysates, whilst others have examined DC-derived exosomes^11,12^ and secretomes^13^. Such studies have provided some insight into the proteomic changes occurring in DCs during the maturation process. However to date, such analyses have been largely qualitative in nature and have only been able to reliably examine a relatively small subset of DC proteins at a time. Also, individual proteins that exhibit altered expression profiles differ greatly between the described reports, with only few proteins in common, limiting the interpretation of the obtained data.

Here we use sequential window acquisition of all theoretical fragment ion spectra mass spectrometry (SWATH-MS), which uses LC-MS/MS for label-free quantitation to describe global proteomic changes in monocyte-derived DCs (moDCs) up to 24 h following lipopolysaccharide (LPS)-induced (TLR4-mediated) maturation. Furthermore, we relate observed proteomic changes to specific cellular pathways. The presented data provides a high degree of quantitative information as to the proteomic and mechanistic changes that occur in moDCs during antigen processing and presentation.

## Results

### Quantitative analysis of the moDC proteome

**Figure 1:**
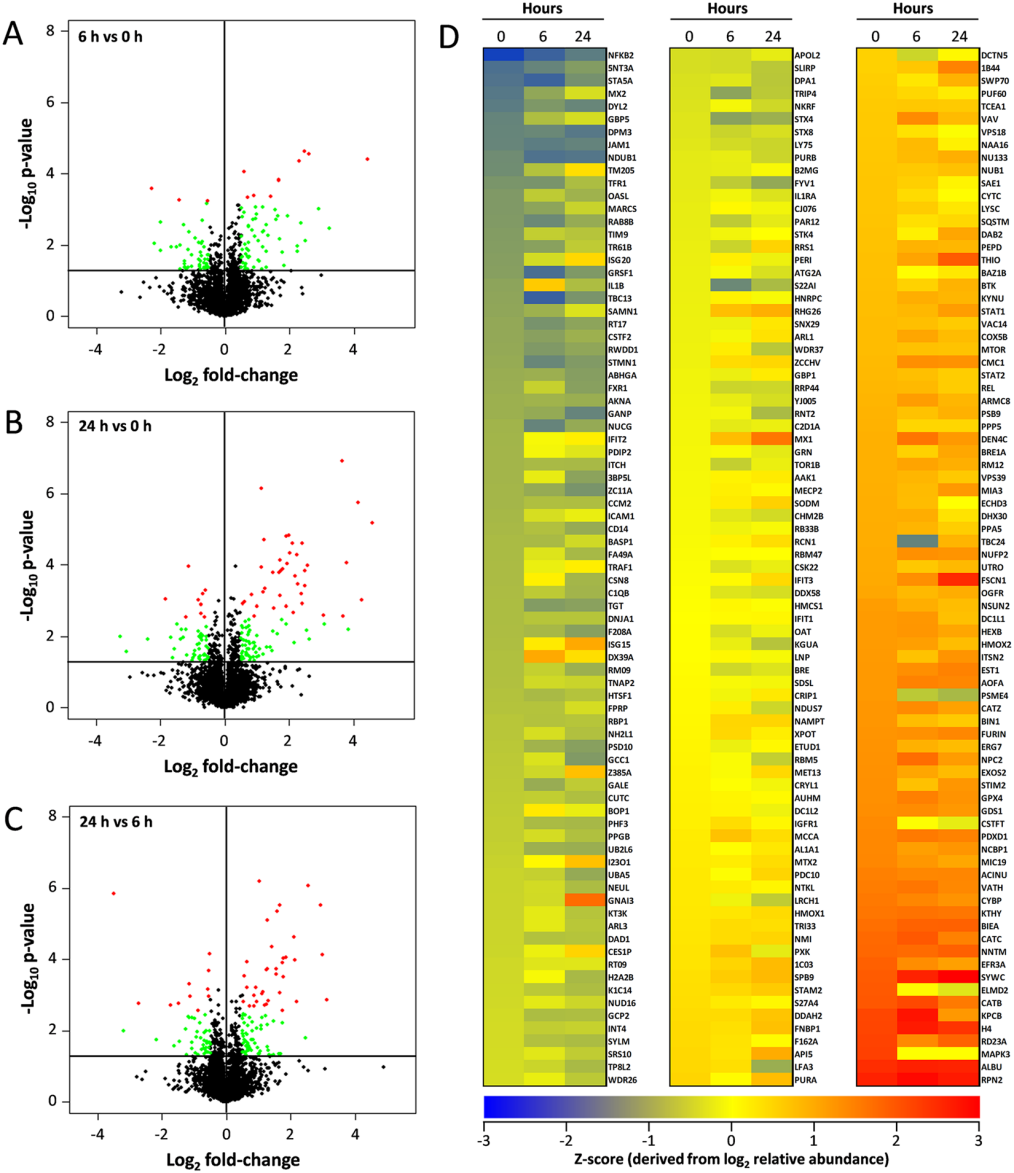
Quantitative proteomic analysis of LPS-mediated moDC maturation over a 24 h period. (**A**–**C**) Volcano plots of all quantified proteins displaying differences in relative abundance at 6 h vs 0 h, 24 h vs 0 h and 24 h vs 6 h, respectively. The plot represents the -log_10_ of the p-value against the log_2_ of the fold change (FC). The green dots represent proteins with p-value ≤ 0.05 and log_2_ fold-change > 0.5. Red dots represent proteins with q-value ≤ 0.2 and log_2_ fold-change >0.5. D. Heat map representing the z-score derived from the log_2_ relative abundance of the total 243 proteins that were significantly up or down-regulated (p-value ≤ 0.05) by >1.5-fold between any two of the three time points (0 h, 6 h or 24 h) examined.

Monocytes, 90–95% CD14+ prior to addition of IL-4 and GM-CSF (not shown), were isolated from blood samples as described in Materials and Methods and differentiated into moDCs^14^. The activation of dendritic cells was assessed using flow cytometry, where the presence of the DC maturation marker, CD83^15^ was confirmed in moDCs from three samples treated with 100 ng/ml LPS. In each case a similar average mean fluorescence upregulation of 3.1-fold was observed following the treatment (Figure S1). In order to generate a spectral library (for use as a reference library to match peptide fragmentation spectra generated in SWATH MS), data-dependent acquisition analysis of the proteomes of untreated moDCs (0 h) and moDCs treated with LPS for 6 and 24 h was performed. This resulted in a reference spectral library consisting of 4,666 proteins with 1% false discovery rate (FDR). To determine the LPS-activation induced changes in the moDC proteome, we quantified the proteins treated with LPS at 0, 6 and 24 h by SWATH-MS. To increase the reliability of our study, proteins quantified based on 2 or more peptides were exclusively selected, this led to selection of 3,494 proteins, relative abundance (denoted by average peak intensity in Table S1) of which were compared at 6 h vs 0 h, 24 h vs 0 h and 24 h vs 6 h. Volcano plots of all 3494 proteins displaying differences in relative abundance at 6 h vs 0 h, 24 h vs 0 h and 24 h vs 6 h are shown in Fig. 1A–C, respectively. Of the 3494 proteins consistently quantified during the time course, the relative abundance of a total of 227 (6.5%) proteins was significantly altered (p-value ≤ 0.05) 6 h after LPS treatment. More profound changes in the proteome were detectable 24 h after LPS treatment, where 287 unique proteins (8.2%) significantly changed (p-value ≤ 0.05). Between 6 h and 24 h a total of 273 unique proteins (7.8%) were significantly changed (p-value ≤ 0.05). Figure 1D shows a heat-map based on z-score (derived from the log_2_ relative abundance) of the total 243 proteins that were significantly altered (p-value ≤ 0.05) by at least 1.5-fold (up or down-regulated) between two of the three time points examined. A post-hoc estimation of FDR (q-value/adjusted p-value) for each of these proteins was additionally performed using Benjamin Hochberg correction. The quantitative information for all 3,494 proteins at peptide level is provided in Table S1. The quantitative value for each unique peptide originates from summing the integrated area of the selected b and y-ions for this peptide and is an average value for each genotypic group (indicated as average peak intensity). All proteins exhibiting statistically significant changes in relative abundance (p-value ≤ 0.05) at 6 h vs 0 h, 24 h vs 0 h and 24 h vs 6 h are listed in Tables S2–S7.

For further functional analysis of differentially regulated proteins, a fold-change cut-off of >1.5-fold was chosen. The SWATH-MS analysis identified 57 proteins that display >1.5-fold change in relative cellular abundance 6 hours after LPS-treatment and 40 proteins were shown to exhibit >1.5-fold reductions (relative change of 0.666) in relative cellular abundance 6 hours after LPS-treatment. Eighty seven proteins were identified to display >1.5-fold change in relative cellular abundance 24 hours relative to 0 h, after LPS-treatment and 46 proteins were shown to exhibit >1.5-fold reductions in relative cellular abundance 24 hours after LPS-treatment. Seventy five proteins were identified to display >1.5-fold change in relative cellular abundance 24 hours relative to 6 h, after LPS-treatment and 39 proteins were shown to exhibit >1.5-fold reductions in relative cellular abundance 24 hours after LPS-treatment.

**Figure 2:**
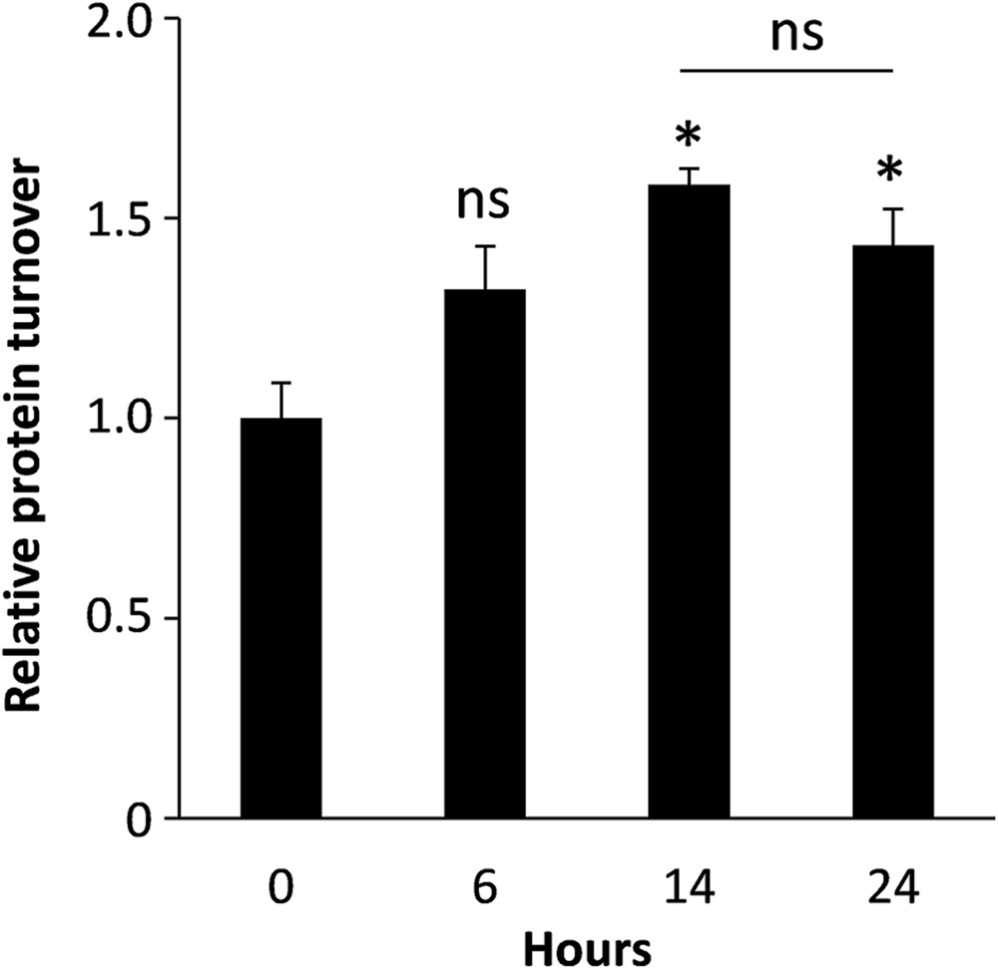
Protein synthesis in LPS-treated moDCs. Protein synthesis was measured using the Click-iT HPG assay kit and results were expressed relative to control (0 h) cells. Error bars represent ± S.E.M. Statistical significance was assessed by t-test (ns: no significant change; *p < 0.05; n = 3).

In addition to the quantitative proteomic analysis, to gain insight into the potential changes in overall protein synthesis at different stages of the maturation process, protein synthesis in moDCs was measured in cells at 0 h, 6 h, 14 h and 24 h after LPS-treatment using the Click-iT HPG assay kit (Fig. 2). Protein synthesis was found to increase by 58% after 14 hours (p ≤ 0.05) relative to the 0 h control. Synthesis was 32% higher than the control after 6 h but this increase was not deemed to be statistically significant. After 24 h protein synthesis was 43% higher than the control (p < 0.05). The difference in protein synthesis observed between 14 h and 24 h was not found to be statistically significant.

### Functional annotation of differentially expressed proteins

Pathway analysis and protein interaction networks for moDC proteins displaying >1.5-fold change; between the respective time points (0 h, 6 h and 24 h) were analysed using the Reactome Pathway Database (https://reactome.org)^16,17^ and by Search Tool for Recurring Instances of Neighbouring Genes (STRING) software (https://string-db.org/)^18^, respectively. The Reactome pathway analysis is an over-representation analysis that statistically determines whether proteins from certain pathways are enriched in the data. This analysis produces a probability score, which is corrected for FDR using the Benjamani-Hochberg method. A summary of the resultant data is shown in Table 1, which details the top 25 pathways ranked by lowest p-value that are upregulated at 6 h relative to 0 h (representing early cellular processes) and at 24 h relative to 0 h (later cellular processes) after LPS stimulation. The full dataset displaying all pathways where the p-value is p < 0.05 for both up- and downregulated proteins (>1.5-fold change) and the respective proteins associated with each can be found in Table S8. Interaction networks for moDC proteins exhibiting the highest degree of significant change (>1.5-fold; p ≤ 0.05) between the respective time points (0 h, 6 h and 24 h) were created using STRING software. STRING is a database of known and predicted protein-protein interactions including direct and indirect associations. Figures S2–4 show all 6 protein interaction networks corresponding to up- or down-regulated proteins between the respective time points (0 h, 6 h and 24 h).

**Table 1:** Summary of Reactome pathway analysis data for MoDC proteins exhibiting statistically significant increases >1.5-fold at 6 h *vs* 0 h and at 24 h *vs* 6 h. The data indicates the pathway, number of proteins found/number of proteins in the pathway, p-value and FDR. The top 25 pathways are shown with proteins in each group ranked by p-value (lowest first).

Protein group	Pathway name	Entities		
		Found	p-value	FDR
Up-regulated; 6 h vs 0 h	Interferon signaling	10/284	2.08E-06	6.69E-04
	Cytokine signaling in the immune system	15/771	7.26E-06	0.001
	Interferon α/β signalling	6/137	8.35E-05	0.009
	Interleukin-1 processing	2/7	6.45E-04	0.031
	DDX58/IFIH1-mediated induction of Interferon-α/β	4/77	7.34E-04	0.031
	ISG15 antiviral mechanism	4/78	7.70E-04	0.031
	Antiviral mechanism by IFN-stimulated genes	4/78	7.70E-04	0.031
	Negative regulators of DDX58/IFIH1 signaling	3/34	7.86E-04	0.031
	Clathrin-mediated endocytosis	4/139	0.006	0.215
	TRAF6-mediated endocytosis	2/24	0.007	0.229
	Termination of translesion DNA synthesis	2/32	0.012	0.278
	Cargo recognition for clathrin-mediated endocytosis	3/99	0.015	0.278
	Biosynthesis of aspirin-triggered D-series resolvins	1/3	0.016	0.278
	Immune system	19/2226	0.016	0.278
	Translesion synthesis by Y family DNA polymerases bypasses lesions on DNA template	2/39	0.018	0.278
	Interleukin-4 and interleukin-13 signaling	3/111	0.021	0.278
	Transcriptional activation of cell cycle inhibitor p21	1/4	0.021	0.278
	Biosynthesis of E-series 18®-resolvins	1/4	0.021	0.278
	Biosynthesis of D-series resolvins	1/4	0.021	0.278
	Interleukin-10 signaling	2/45	0.023	0.278
	PKMTs methylate histone lysines	2/47	0.025	0.278
	Biosyntheis of E-series 18(S)-resolvins	1/5	0.026	0.278
	DNA damage bypass	2/49	0.027	0.278
	Synthesis of 15-eicosatetraenoic acid derivatives	1/6	0.031	0.278
Up-regulated; 24 h vs 6 h	Antigen presentation: Folding, assembly and peptide loading of class I MHC	50/93	1.11E-16	4.22E-15
	Endosomal/vaculolar pathway	50/79	1.11E-16	4.22E-15
	Interferon signaling	63/284	1.11E-16	4.22E-15
	Interferon gamma signaling	54/172	1.11E-16	4.22E-15
	Class I MHC mediated antigen processing and presentation	54/440	1.11E-16	4.22E-15
	ER phagosome pathway	52/152	1.11E-16	4.22E-15
	Interferon α/β signaling	57/137	1.11E-16	4.22E-15
	Antigen processing-cross presentation	52/168	1.11E-16	4.22E-15
	Immuno-regulatory interactions between a lymphoid and a non-lymphoid cell	51/297	1.11E-16	4.22E-15
	Cytokine signaling in immune system	68/771	1.11E-16	4.22E-15
	Adaptive immune system	56/944	1.11E-16	4.22E-15
	Immune system	72/2226	1.11E-16	4.22E-15
	DAP12 interactions	5/46	1.92E-04	0.007
	TBC/RABGAPs	4/45	0.002	0.057
	Antiviral mechanism by IFN-stimulated genes	5/78	0.002	0.057
	ISG15 antiviral mechanism	5/78	0.002	0.057
	Interleukin-9 signaling	2/9	0.005	0.124
	Interleukin-21 signaling	2/10	0.006	0.147
	Defective GALE can cause epimerase-deficiency galactosemia (EDG)	1/1	0.011	0.247
	Defective SLC27A4 causes ichtyosis prematurity syndrome	1/1	0.011	0.247
	Interleukin-6 signaling	2/15	0.013	0.267
	Signaling by SCF-KIT	3/44	0.014	0.273
	The NLRP inflammasome	2/16	0.014	0.273
	Signalling by cytosolic FGFR1 fusion	2/18	0.018	0.341
	Signaling by PDGF	3/59	0.03	0.487

### Changes in cytokines and cytokine-induced proteins in LPS-stimulated moDCs

**Figure 3:**
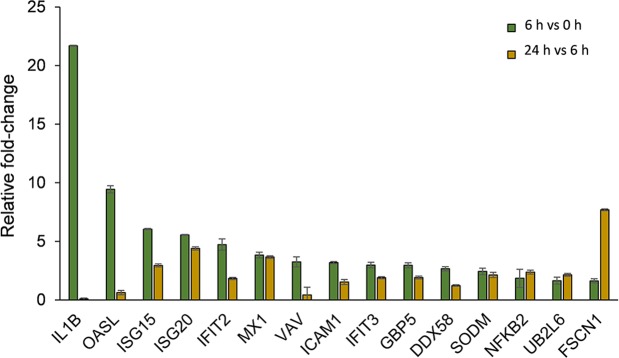
LPS-induced changes in moDC proteins associated with cytokine signaling. Comparison of the relative fold-changes in cellular abundance of cytokine signaling proteins in moDCs at 6 *vs* 0 h and 24 h *vs* 6 h post-LPS stimulation as measured by SWATH-MS. Error bars represent ± S.E.M.

It was apparent from the pathway analyses that a number of moDC proteins associated with cytokine signalling and in particular, interferon and interleukin signalling had increased in expression following LPS treatment over the time course. Quantitative data relating to key proteins implicated in these pathways are shown in Fig. 3. The relative abundance of the cytokine, IL-1β was dramatically increased 21.7-fold) at 6 h post-treatment, but decreased after 24 h. A number of known interferon-responsive proteins were found to exhibit large increases in expression over the course of the experiment, particularly in the period up to 6 h post-stimulation. These included guanylate-binding proteins GBP5, interferon-induced GTP-binding proteins, Mx1 and Mx2, interferon-induced proteins with tetratricopeptide repeats, IFIT2, IFIT3 and interferon-stimulated gene proteins, ISG-15 and ISG20. The related OASL protein, exhibited a 9-fold increase in relative abundance at 6 h and remained highly expressed, albeit at slightly lower levels at 24 h (6-fold relative to 0 h control). The interferon-responsive ubiquitin-conjugating enzyme, UB2L6 exhibited 1.6 and 3.5-fold increases in expression after 6 and 24 h, respectively. The cytokine-responsive cytoskeletal protein, fascin (FSCN1) underwent a large increase in expression (7.7-fold) between 6–24 h.

### Changes in endocytic/phagocytic and MHC proteins in LPS-stimulated moDCs

**Figure 4:**
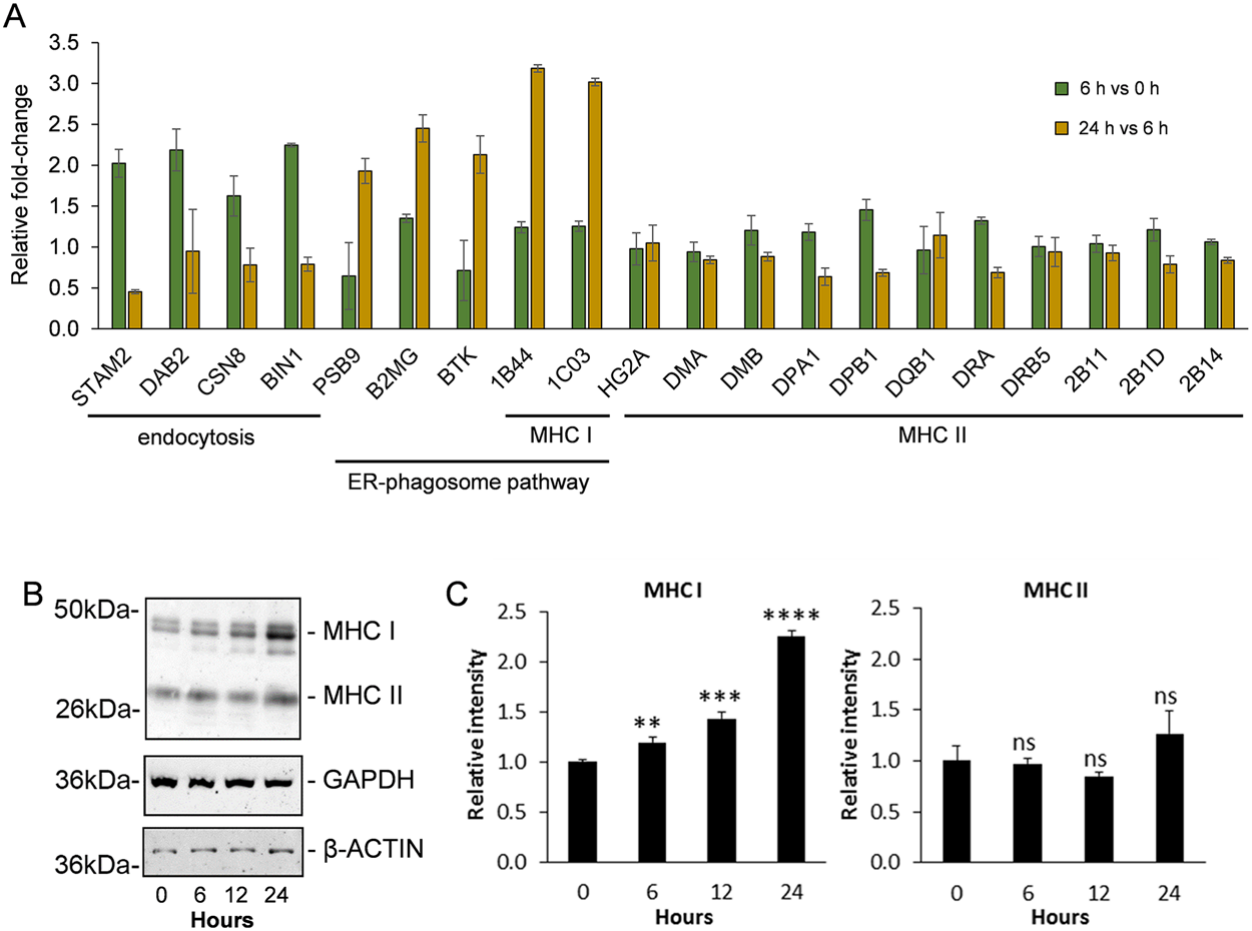
LPS-induced changes in endocytic/phagocytic and MHC proteins in moDCs. (**A**) Comparison of the relative fold-change in cellular abundance of endocytic/phagocytic and MHC proteins in moDCs at 6 *vs* 0 h and 24 h *vs* 6 h post-LPS stimulation as measured by SWATH-MS. Error bars represent ± S.E.M. (**B**) Western blot showing relative changes in MHC I and II proteins in moDCs between 0–24 h after LPS stimulation. (**C**) Quantification of MHC I and II proteins based on densitometry analysis of bands in (**B**). Protein levels were calculated relative to the 0 h control. Error bars represent ± S.E.M. Statistical significance was assessed by t-test (ns: no significant change; **p < 0.01; ***p < 0.001; ****p < 0.0001; n = 3).

For extracellular antigens to be processed prior to presentation by MHC molecules they must first enter the cell via an endocytic or phagocytic mechanism. Reactome pathway analysis revealed 4 proteins involved in clathrin-mediated endocytosis, to be upregulated by >1.5-fold early in the maturation process (by 6 h post LPS-stimulation). These were signal transducing adapter molecule 2 (STAM2), disabled homolog 2 (DAB2), COP 9 signalosome complex subunit 8 (CSN8) and myc box-dependent-interacting protein 1 (BIN1). Three of which, STAM2, DAB2 and CSN8, are involved in cargo recognition. Later in the maturation process it was revealed that 5 proteins involved in ER-phagosome recycling were upregulated at 24 h relative to 6 h. These proteins were proteasome subunit beta type-9 (PSB9), beta-2-microglobulin (B2MG), tyrosine protein kinase BTK (BTK) and the two MHC I molecules, HLA class I histocompatibility antigens B-44 alpha chain and Cw-3 alpha chain (1B44 and 1C03, respectively). Contrary to this, it appeared that a number of proteins associated with MHC class II antigen presentation decreased between 6 h and 24 h. An examination of all MHC proteins detected and quantified by SWATH-MS revealed data pertaining to two MHC I proteins (those indicated above) and eleven MHC II proteins (Fig. 4A). When compared to the levels present in the 0 h control, both MHC I proteins detected and quantified (1B44 and 1C03) were found to be significantly more abundant 24 h after treatment (by 3.9-fold and 3.7-fold vs 0 h control, respectively), having undergone the largest increase in expression between 6 h to 24 h. Of the eleven MHC II proteins detected and quantified none exhibited any particularly large changes in relative abundance at 6 h or 24 h after LPS treatment relative to the 0 h control, although most displayed modest reduction at 24 h relative to 6 h. To further examine changes in expression of MHC proteins, Western blots were performed on moDC extracts following 0, 6, 12 and 24 h of LPS treatment using antibodies that specifically recognized MHC class I and MHC class II molecules, as well as β-actin and glyceraldehyde 3-phosphate dehydrogenase (GAPDH) as loading controls (Fig. 4B). A densitometric analysis of the resultant bands revealed that the collective expression of MHC class I molecules significantly increased over the course of the experiment (>2-fold), whilst MHC class II expression remained relatively constant (Fig. 4C).

## Discussion

This study is the first to use SWATH-MS to quantify global proteomic changes occurring in moDCs during LPS-stimulated maturation. In this study 3,494 cellular proteins (which were quantified based on 2 or more peptides) were analysed at 0 h, 6 h and 24 h after stimulation. Collectively, the results agreed with and vastly built upon other proteomic studies focussed on moDC maturation. For example, previously, using a shotgun proteomics approach, Gundacker *et al*. identified potential changes in 22 moDC proteins (based on the relative number of peptides per protein) in response to LPS after 24 h^13^. Of these 22 proteins, 11 were also shown in our study to display statistically significantly increased relative abundance in moDCs and many of these (such ISG20, ISG15, Mx2, Mx1, IFIT2, IFIT3, ICAM1 and GBP1) were among those that exhibited the greatest increases in relative cellular abundance after 24 h. More recently, Strasser *et al*. carried out a study examining proteomic changes in moDCs treated with LPS as well as birch pollen allergens for 8 h and compared differences in protein expression between treatments^19^. In their study they used high-performance liquid chromatography and tandem mass spectrometry (HPLC-MS/MS) following isobaric labelling of the samples for proteomic quantification. Whilst their specific study question was different from ours, they were able to identify expression of a large number of proteins also observed in our study.

Pathway analysis of proteins exhibiting >1.5-fold changes in relative abundance between the different time points (0 h, 6 h and 24 h) revealed clusters of related proteins involved in specific cellular pathways and functions. Of all proteins that significantly changed at 24 h relative to 0 h, ~39% displayed a decrease in relative abundance. This suggests that reducing the degree to which all but essential proteins and compounds are synthesized appears to be a strategy the cell uses to reduce its metabolic burden. Generally, fewer interactions were observed between decreasing proteins in the respective STRING analyses for proteins that increased in relative abundance between the three respective time points compared to increasing proteins. Reactome pathway database for these decreasing proteins (>1.5-fold reduction) led to significant pathway hits, which varied between time points (although these tended to have higher FDR and p-values compared with the corresponding upregulated protein results). Early in the maturation process (0 h to 6 h) it was found that pathways related to mitochondrial protein import and cristae formation as well as synthesis of phosphatidylinositol phosphates at the endosomal membrane were among the most significant hits. Later in the maturation process (between 6 h to 24 h) the most significant downregulated pathway was MHC class II antigen presentation, reflecting the observed reductions in relative abundance of MHC II molecules over this period.

In the first 6 h after LPS-mediated activation the most significant pathways identified for upregulated proteins related to cytokine (interferon and interleukin) signalling, endocytosis and the synthesis of resolvins, lipid mediators that promote restoration of function after inflammation. Between 6 h to 24 h after LPS stimulation, representing a later stage in the moDC maturation process, pathways associated with antigen-presentation were among the most significant hits. Over this period, pathways associated interferon signalling (α/β and γ-associated subtypes) were significantly upregulated (FDR = 4.22 × 10^−14^) as were interleukin signalling pathways.

The cytokine which displayed the greatest degree of change in response to LPS was IL-1β, which exhibited a 22-fold increase in relative abundance by 6 h, in agreement with other studies^20,21^. STRING analysis revealed IL-1β, a key initiator of several pathways early in the dendritic cell maturation process, to be a central protein in the interaction network through linking to proteins involved in signal transduction and cellular responses to (oxidative) stress. A key cluster in the STRING network stemming from IL-1β is a group of proteins involved in interferon signalling, which linked to (most likely due to direct activation of) various clusters of proteins. One such cluster contained proteins involved in protein synthesis, which include ribosome biogenesis regulatory protein homolog (RRS1) and elongation factor Tu GTP-binding domain-containing protein 1 (ETUD1). This was potentially in agreement with the observation that protein synthesis in LPS-stimulated moDCs increased over the first 14 h. After 24 h of LPS treatment, the relative cellular abundance of IL-1β in moDCs was found to drop to almost basal levels, suggesting that essentially all of what is synthesized by 6 h is released and/or degraded over this period. IL-1 cytokines are secreted by the non-classical secretory pathway and require to be released by independent signals. Treatment of bone marrow-derived DCs with LPS and ATP has been shown to trigger IL-1β secretion via the P2X7 receptor^22^. Cytosolic IL-1 proteins have been shown to undergo ubiquitination, which was previously demonstrated to be a central mechanism for the regulation of intracellular IL-1 levels^23^. Consistent with this, >1.5-fold increases in the expression of ubiquitin function-related enzymes, UB2L6 (ubiquitin conjugating enzyme E2 L6) and UBA5 (ubiquitin-like modifier activating enzyme 5) were observed between 6 h and 24 h after LPS stimulation.

IFN-αβ is known to be produced by DCs, while IFN-γ is an established autocrine mediator of DC maturation and is produced and secreted by LPS-stimulated bone marrow-derived DCs within 24 h of activation^24^. Over the course of the 24 h after LPS treatment the relative abundance of multiple proteins involved in cytokine/interferon signal transduction were found to change in moDCs. The SWATH-MS analysis was unable to verify expression of IFNs directly but revealed profound increases in the expression of various IFN-responsive proteins, particularly between 6 and 24 h post stimulation. Microarray studies have previously identified up to 1,000 IFN-sensitive genes, with 200–500 genes typical of many cell types^25–27^. Many of these proteins function to inhibit the multiplication of viruses by inhibiting viral DNA replication^28^; these include interferon-induced binding proteins Mx1, interferon-induced protein with tetratricopeptide repeats 3 (IFIT3), guanylate binding protein 5 (GBP5) and 2′-5′-oligoadenylate synthase-like enzyme, which were observed in this study to display increased expression in moDCs in response to LPS stimulation.

For moDC-associated antigen-processing to occur, cellular detection and endocytic/phagocytic entry of antigen into the cell is required prior to presentation by MHC-II molecules (or cross-presentation by MHC-I molecules). In our study a number of endocytic proteins including cargo-recognition proteins were found to be upregulated following LPS stimulation, particularly by 6 h, consistent with endocytic/phagocytic entry of antigen into the cell. Later in the maturation process LPS-stimulation led to relative increases in ER-phagosome pathway proteins and MHC I proteins in moDCs after 24 h (as was reflected in the pathway analyses for upregulated proteins at 24 h relative to both 0 h and 6 h). The ER-phagosome pathway is a mechanism moDCs use where part of the ER membrane is recruited to the cell surface and fuses with the plasma membrane. Underneath this portion of the membrane are phagocytic cups, which are thought to supply membrane for the formation of nascent phagosomes^29^. MHC I molecules generally present peptides that are derived from endogenous proteins present in the cytosol but in some DC sub-types (including moDCs) they can cross-present extracellular antigens^30,31^. LPS-stimulation had a comparatively modest effect on the expression of MHC II molecules in moDCs, inducing only negligible differences in expression (<1.2-fold) in the MHC II proteins detected. No difference in band intensity could be seen by Western blotting when using an antibody that recognizes MHC II proteins. However, consistent with our data, it is thought that at steady state, the rate of generation of MHC II-peptide complexes in immature DCs is approximately equal to the rate of their degeneration^32,33^, with their exposure at the plasma membrane regulated by ubiquitination^32,34^.

LPS-mediated moDC cell maturation is known to induce a change in cellular morphology, characterized an increase in filamentous actin and the appearance of veils in mature cells^35^. This change in morphology becomes evident in cultured moDCs by 6 h, and continues beyond 24 h after LPS-stimulation^35^, this was also supported by another study which showed that LPS-induced maturation leads to robust upregulation of the actin-bundling protein, fascin^36^. In accordance with these reports, proteins related to filamentous actin assembly were found to be differentially expressed in our SWATH-MS study. Fascin (FSCN1) increases greatly between 6 h and 24 h post-LPS treatment. Similarly, cytoplasmic actin (ACTB) increases by 1.5-fold at 6 h and remains around this level at 24 h. It has already been demonstrated that the F-actin network in LPS stimulated dendritic cells, regulates the lateral mobility of intercellular adhesion molecule 1 (ICAM1)^37^. Interestingly, intercellular adhesion molecule 1 (ICAM1) was consistently upregulated upon LPS-stimulation at 6 h and 24 h relative to the 0 h control. Whilst there were some differences in the observed expression of actin cytoskeletal regulatory proteins, very little change was observed in proteins related to tubulin assembly. Over the course of the 24 h after stimulation there were modest increases in tubulin beta-8 chain (TBB8), tubulin-folding cofactor B (TBCB) and gamma-tubulin complex component 3 (GCP3) at various points but all of which were of a lower fold-change than our cut-off for cluster analysis by Reactome/STRING.

In conclusion, this study further supports SWATH-MS as a robust technology for the quantitative study of proteins involved in cellular processes, such as is demonstrated here for pathogenic stimulation of moDCs. The data presented provides the most detailed insight into the proteomic changes that occur during moDC maturation to date and greatly builds on previous proteomic studies. LPS-mediated activation was found to lead to a significant change in the relative cellular abundance of around 14.5% of quantified moDC proteins. Specifically, relative abundance of proteins involved in interferon and interleukin signalling, endocytosis, the ER-phagosome pathway and antigen-presentation are significantly altered in the moDCs following LPS stimulation. The upregulation of proteins that contribute to these pathways is characterized by an observable corresponding increase in protein synthesis during the same period. We believe that this dataset will provide as a useful resource to others interested in the study of DCs and other antigen-presenting cells.

## Materials and Methods

### Ethical approval and informed consent

This study was approved by the School of Medicine Ethics Committee, University of St Andrews. In all cases blood samples were taken after obtaining written informed consent from the donors or from commercially obtained buffy coat preparations, as indicated. All methods outlined were performed in accordance with the relevant ethical guidelines and regulations.

### Culture of monocyte-derived DCs

For flow cytometry experiments, blood was collected from two healthy donors and one commercial buffy coat donor. For SWATH-MS, blood was obtained from four healthy donors and pooled in various combinations to form three biological replicates. Samples were purified over a ficoll gradient at room temperature to isolate peripheral blood mononuclear cells (PBMCs; Histopaque, Sigma-Aldrich, Poole, UK). PBMCs were plated for 30–60 mins, non-adherent cells removed and the remaining monocytes differentiated into moDCs as described previously using IL-4 and GM-CSF^14^. The moDCs were treated with 100 ng/ml of bacterial LPS (Sigma-Aldrich), or left untreated as control. Cells were cultured in sterile RPMI containing 10% fetal calf serum for up to 24 h.

### Flow cytometry

To examine expression of the moDC maturation marker, CD83, moDCs were stimulated with 100 ng/ml LPS for 24 hours and stained with mouse anti-human CD83 antibody (Serotec MCA1582F). Analysis was performed using a Guava 8HT flow cytometer (Merck-Millipore UK) running GuavaSoft 2.7 software.

### Cell lysis and protein quantification

Cells were washed on ice with phosphate buffered saline (PBS) and lysed for 30 min at 55 °C with 300 µl of lysis buffer (10 mM Tris-Cl, 150 mM NaCl, 0.5% Rapi Gest, pH 7.9). Cells were further lysed by passaging through a syringe. Cell debris was removed following centrifugation and the resultant proteins were quantified using the bicinchoninic acid (BCA) protein assay kit (Thermo Fisher Scientific, Perth, UK).

### Experimental design and statistical rationale for SWATH-MS

This experiment uses untreated dendritic cells (0 h) as control samples for basal protein expression levels. Experiments were performed in biological triplicate. To account for the probability of minor sample variability due to multiple steps in sample processing, one sample at each time point was ran as a technical replicate. A principal component analysis of the technical replicates showed excellent agreement between the resultant datasets (Figure S5). A sample-specific library was made by pooling all conditions for best sample representation.

### Sample preparation for mass spectrometry

Two sets of tryptic digests of samples were prepared: Set 1 (library) consisted of 170 μg of each protein sample combined to yield 1500 µg of protein to be further fractionated by strong cation exchange (SCX) chromatography and high pH reversed phase chromatography. In Set 2, 30 μg of each sample was digested separately for SWATH analysis. The same digestion procedures were carried out on all samples (the combined set 1 and the individual samples in set 2). To denature the protein, a stock solution of 10 M urea in 50 mM ammonium bicarbonate was prepared and used to adjust all samples to a final concentration of 5 M urea. Proteins were reduced and alkylated with 5 mM tris (2-carboxyethyl) phosphine followed by 5 mM iodoacetamide. The reaction was quenched with 10 mM dithiothreitol. Samples were diluted with 50 mM ammonium bicarbonate to a final urea concentration of 1.5 M. The resulting samples were then digested with trypsin (1:50 ratio (w/w), 0.2 µg/μl trypsin; Promega, Southampton, UK), overnight at 30 °C. To stop the digestion, 0.5% (v/v) trifluoroacetic acid (TFA) was added. Peptides were desalted using a C18 SepPak cartridge (Waters, Elstree, UK) and the solvent removed using a SpeedVac (Thermo Fisher Scientific).

### LC-ESI-MSMS analysis for spectral library generation

Once re-dissolved in 1 ml of 10 mM ammonium formate, 25% acetonitrile (MeCN), pH 3.0, 800 μg of peptides from the combined sample (set 1) were fractionated by SCX chromatography on a PolySulfoethyl A column (2.1 mm × 200 mm, 5 µm, 200 Å pore size, PolyLC). The column was washed with 100% Buffer Ascx (10 mM ammonium formate, 25% MeCN, pH 3.0) at 1 ml/min for 22 min. A linear gradient of 0–50% Bscx (500 mM ammonium formate, 25% MeCN, pH 3.0) was applied over 20 min, 50–100% Bscx over 3 min, followed by 100% Bscx for a further 3 min to wash the column, before re-equilibration in 100% Ascx for another 11 min. Fractions of 0.5 ml were collected every 30 s. The UV chromatogram was inspected and fractions pooled to give 7 fractions across the elution profile. The pooled fractions were dried and dissolved in 0.1% formic acid (FA). They were desalted on C18 spin columns (PepClean C18 spin columns, ThermoScientific) using the manufacturer’s instructions, eluting in 60 μl 70% MeCN/0.5% TFA. The elution solvent was removed in a SpeedVac and the fractions resuspended in 20 μl 0.1% FA prior to mass spectrometric analysis.

For high pH reversed phase fractionation, 650 µg of peptides (remainder of set 1) were resuspended in 100 µl Buffer A, consisting of 10 mM ammonium formate, 2% MeCN, pH 10.0. Peptides were then fractionated on a XBridge C18 column (4.6 × 100 mm, 5 µm, Waters) at 1 ml/min with the following gradient: linear gradient of 4–28% Buffer B (10 mM ammonium formate, 90% MeCN, pH 10.0) for 36 min, then 28–50% B for 8 min, followed by 100% B for a further 5 min to wash the column, before re-equilibration in 100% A for 10 min. Fractions of 0.5 ml were collected every 30 s. The UV chromatogram was inspected and fractions pooled to give 10 fractions across the elution profile. The pooled fractions were dried and resuspended in 0.1% FA for mass spectrometric analysis.

For spectral library generation, each SCX fraction (1/3 of vol) and each high pH reversed phase fraction (1/3 of volume) were analysed individually on a Sciex TripleTOF 5600+ system mass spectrometer (Sciex, Framingham, MA, USA) coupled to an Eksigent nanoLC AS-2/2Dplus system, in data dependent mode, to achieve in depth identification of proteins. Additionally 1 μg of peptides from each individually digested sample (set 2) were combined and also analysed in data dependent mode. Prior to mass spectrometric analysis, reference iRT peptides (Biognosys, Schlieren, Switzerland) were added to each sample according to the manufacturer’s specifications to allow correction of retention times. The samples were loaded in loading buffer (2% MeCN, 0.05% trifluoroacetic acid) and bound to an Acclaim Pepmap 100 µm × 2 cm trap (Thermo Fisher Scientific), and washed for 10 min to waste, after which the trap was turned in-line with the analytical column (Acclaim Pepmap RSLC 75 µm × 15 cm). The analytical solvent system consisted of Buffer A (2% MeCN, 0.1% FA in water) and Buffer B (2% water, 0.1% FA in MeCN) at a flow rate of 300 nl/min, with the following gradient: linear 1–20% of Buffer B over 90 min, linear 20–40% of Buffer B over 30 min, linear 40–99% of Buffer B over 10 min, isocratic 99% of Buffer B for 5 min, linear 99–1% of buffer B over 2.5 min and isocratic 1% solvent buffer B for 12.5 min. The mass spectrometer was operated in data-dependent analysis (DDA) top 20 positive ion mode, with 250 and 150 ms acquisition time for the MS1 (m/z 400–1200) and MS2 (m/z 230–1800) scans respectively, and 15 s dynamic exclusion. Rolling collision energy with a collision energy spread of 5 eV was used for fragmentation. One search result was generated from raw.wiff files, by merging the combined sample’s DDA data, 7 SCX fractions and 10 high pH reversed phase DDA data, using Protein Pilot v5.0.1 (Sciex) with the following search parameters: urea denaturation as special factors, trypsin as the cleavage enzyme (/K-\P and /R-\P) and carbamidomethylation as a fixed modification of cysteines. Within the TripleTOF 5600+ instrument setting option, MS tolerance was pre-set to 0.05 Da and MS/MS tolerance to 0.1 Da. The search was carried out in “rapid ID” mode with a detected protein threshold of 1% plus false discovery rate analysis against the SwissProt database downloaded June 2015, containing only proteins from humans (40408 proteins). Note that the iRT peptides were included in this database.

### SWATH-MS data acquisition

For SWATH-MS data acquisition, the same mass spectrometer and LC-MS/MS setup was used essentially as described above, but operated in SWATH mode. The method uses 50 windows of variable Da effective isolation width with a 1 Da overlap using Sciex Variable Window Calculator tool. Each window has a dwell time of 150 ms to cover the mass range of 400–1250 m/z in TOF-MS mode and MS/MS data is acquired over a range of 230–1800 m/z with high sensitivity setting and a dwell time of 70 ms, resulting in a cycle time of 3.6 s. The collision energy for each window was set using the collision energy of a 2+ ion centred in the middle of the window with a spread of 5 eV.

### Data processing and availability

Identified proteins within Protein Pilot with a 1% FDR were imported into Skyline Daily 4.1.1.11725^38^ for spectral library generation. SWATH-MS results were mapped against this library. Skyline parameters were chosen as follows: Peptide settings (trypsin digestion with 1 missed cleavage, human as background proteome, carbamidomethylation (C) and oxidation (M) as modifications); Transition settings (2^+^ and 3^+^ precursor charges, 1^+^ and 2^+^ ion charges, b and y ions and precursor. Six transitions were selected per peptide. Final peak area was normalized based on the median value and then processed using statistical tests in skyline software (student’s t-test for p-value and Benjamini Hochberg method for adjusted p-value/q-value). The mass spectrometry proteomics data have been deposited to the ProteomeXchange Consortium via the PRIDE^39^ partner repository with the dataset identifier PXD008898.

### Protein synthesis assay

Quantification of newly synthesized proteins was performed using the Click-iT HPG assay kit (Thermo Scientific). MoDCs were transferred to clear bottom 96-well cell culture plates at 105 cells/well and treated with 100 ng/ml of LPS for up to 24 h. The assay was then performed according to manufacturer’s instruction using RPMI L-methionine-free medium as a solvent for kit components. As a negative control, cells were treated with 35 µM cyclohexamide for up to 24 h. In summary, a methionine analogue, L-homopropargylglycine (HPG) is added to the culture media and incorporated into proteins during active protein synthesis. For detection of the incorporated amino acid Alexa Fluor 488 azide was used, which reacts with the alkyne-modified protein in a chemo-selective manner. For cell quantification, nuclear staining using the HCS Nuclear Mask Blue Stain (Thermo Scientific) was utilised.

### Western blotting

MoDCs were cultured on 12-well plates and stimulated with 100 ng/ml of LPS for up to 24 h. Non-adherent cells were isolated and lysed, and the remaining adherent cells were washed twice with PBS, followed by the addition of 200 μl of RIPA (radioimmunoprecipitation assay) buffer (Cell Signaling Technology, Danvers, MA, USA). Cells were scraped and then lysed for an additional 10 min, at 4 °C, and pooled with the non-adherent lysates. The lysates were centrifuged at 20,000 × g for 5 mins and the resultant supernatants measured using the BCA assay. The samples were then suspended in reducing buffer and denatured for 60 min at 60 °C. Proteins were separated by 10% sodium dodecyl sulfate polyacrylamide gel electrophoresis and then finally transferred to Immobilon-P PVDF membranes (Millipore, Hertfordshire, UK: cat no. IPVH00010). After transfer, the membranes were blocked in LiCor Blocking Buffer (diluted 50:50 in PBS) for 1 h at room temperature before incubating with primary antibodies overnight at 4 °C. Antibodies used were as follows: MHC I (HC10, anti-HLA-B/C, used at 1:1000), MHC II (Abcam, Cambridge, UK; ab157210, used at 1:1000) and β-actin (Santa Cruz, sc-47778; used at 1:1000), GAPDH (Santa Cruz sc-47724, used at 1:1000). Detection was then carried out by first washing membranes in PBS containing 0.1% Tween20 (PBST; 3 × 5 min) followed by a 45 min incubation at room temperature with IRDye800cw-labeled goat anti-mouse or -rabbit IgG secondary antibody (Li-Cor Biosciences, Cambridge, UK) at 1:10,000 dilution in PBST. After further washing with and PBS (1 × 5 min), visualization was carried out using a LiCor Odyssey Scanner (Li-Cor Biosciences).

## Supplementary information

Supplementary Table S1

Supplementary Figures S1-S5 and Tables S2-S7

Supplementary Table S8
