# Supplementary material for: Quantitative proteomic changes in LPS-activated monocyte-derived dendritic cells: A SWATH-MS study
Source: Sci Rep. 2019 Mar 13;9:4343. doi: 10.1038/s41598-019-40773-6 (PMC6416353; doi:10.1038/s41598-019-40773-6)
Supplement: Supplementary file 2 — Supplementary Figures S1-S5 and Tables S2-S7 [file 41598_2019_40773_MOESM2_ESM.pdf]

Supplementary Information

Quantitative proteomic changes in LPS-activated monocyte-derived dendritic cells: A SWATH-MS study

Swati Arya*[1,2], Dagmara Wiatrek-Moumoulidis*[1,2], Silvia A. Synowsky[2], Sally L. Shirran[2], Catherine H. Botting[2], Simon J. Powis[1,2] & Alan J. Stewart[1,2]

[1]School of Medicine, University of St Andrews, St Andrews, KY16 9TF, UK.

[2]Biomedical Sciences Research Complex, University of St Andrews, St Andrews, KY16 9ST, UK.

List of figures and tables

Figure S1: CD83 expression on moDCs as analysed by flow cytometry.

Figure S2: String analyses of dendritic cell proteins displaying significant (p<0.05) changes in cellular abundance (>1.5-fold) upregulation between 0 and 6 h after LPS-stimulation.

Figure S3: String analyses of dendritic cell proteins displaying significant (p<0.05) changes in cellular abundance (>1.5-fold) upregulation between 0 and 24 h after LPS-stimulation.

Figure S4: String analyses of dendritic cell proteins displaying significant (p<0.05) changes in cellular abundance (>1.5-fold) upregulation between 6 and 24 h after LPS-stimulation.

Figure S5: Principal component analysis of SWATH-MS data from technical and biological replicates of moDCs at 0 h, 6 h and 24 h after LPS treatment.

Table S2: Proteins significantly upregulated (p-value≤0.05) at 6 h as compared to 0 h post LPS-stimulation.

Table S3: Proteins significantly downregulated (p-value≤0.05) at 6 h as compared to 0 h post LPS-stimulation.

Table S4: Proteins significantly upregulated (p-value≤0.05) at 24 h as compared to 0 h post LPS-stimulation.

Table S5: Proteins significantly downregulated (p-value≤0.05) at 24 h as compared to 0 h post LPS-stimulation.

Table S6: Proteins significantly upregulated (p-value≤0.05) at 24 h as compared to 6 h post LPS-stimulation.

Table S7: Proteins significantly downregulated (p-value≤0.05) at 24 h as compared to 6 h post LPS-stimulation.

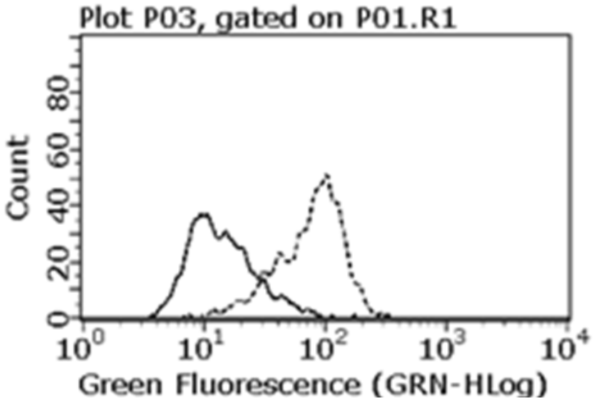

Figure S1. CD83 expression on moDCs as analysed by flow cytometry. moDCs were incubated with or without 100 ng/ml LPS for 24 hours and stained with anti-CD83-FITC. Representative data shown for 100 ng/ml LPS from one of three moDC samples.

6 hours vs 0 hours

Upregulated proteins **Downregulated proteins**

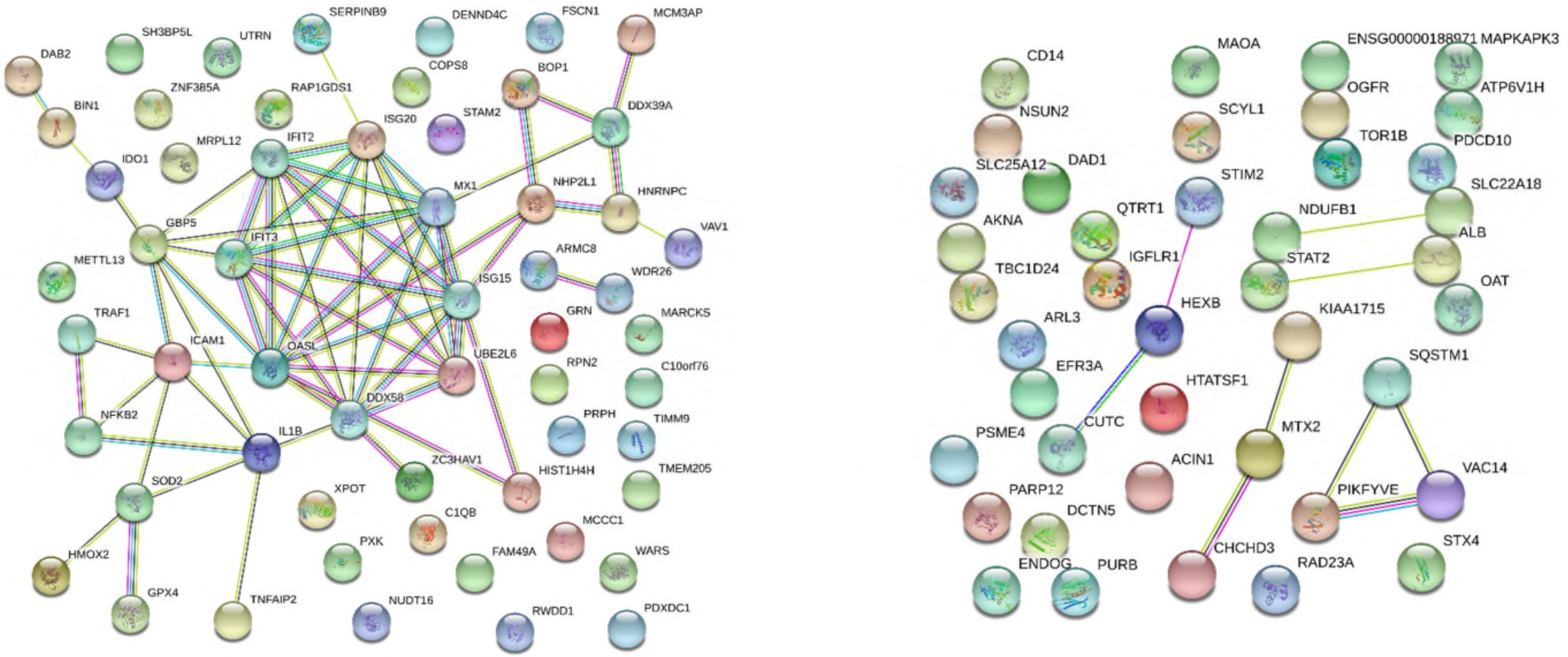

Figure S2. String analyses of dendritic cell proteins displaying significant ($p<0.05$) changes in cellular abundance (>1.5-fold) upregulation between 0 and 6 h after LPS-stimulation. Networks were drawn with seven differently coloured lines: green lines indicate neighbourhood evidence; red lines indicate the presence of gene fusion evidence; blue lines indicate co-occurrence evidence; purple lines indicate experimental evidence; yellow lines indicate text-mining evidence; light blue lines indicate database evidence; and a black line indicates co-expression evidence.

24 hours vs 0 hours

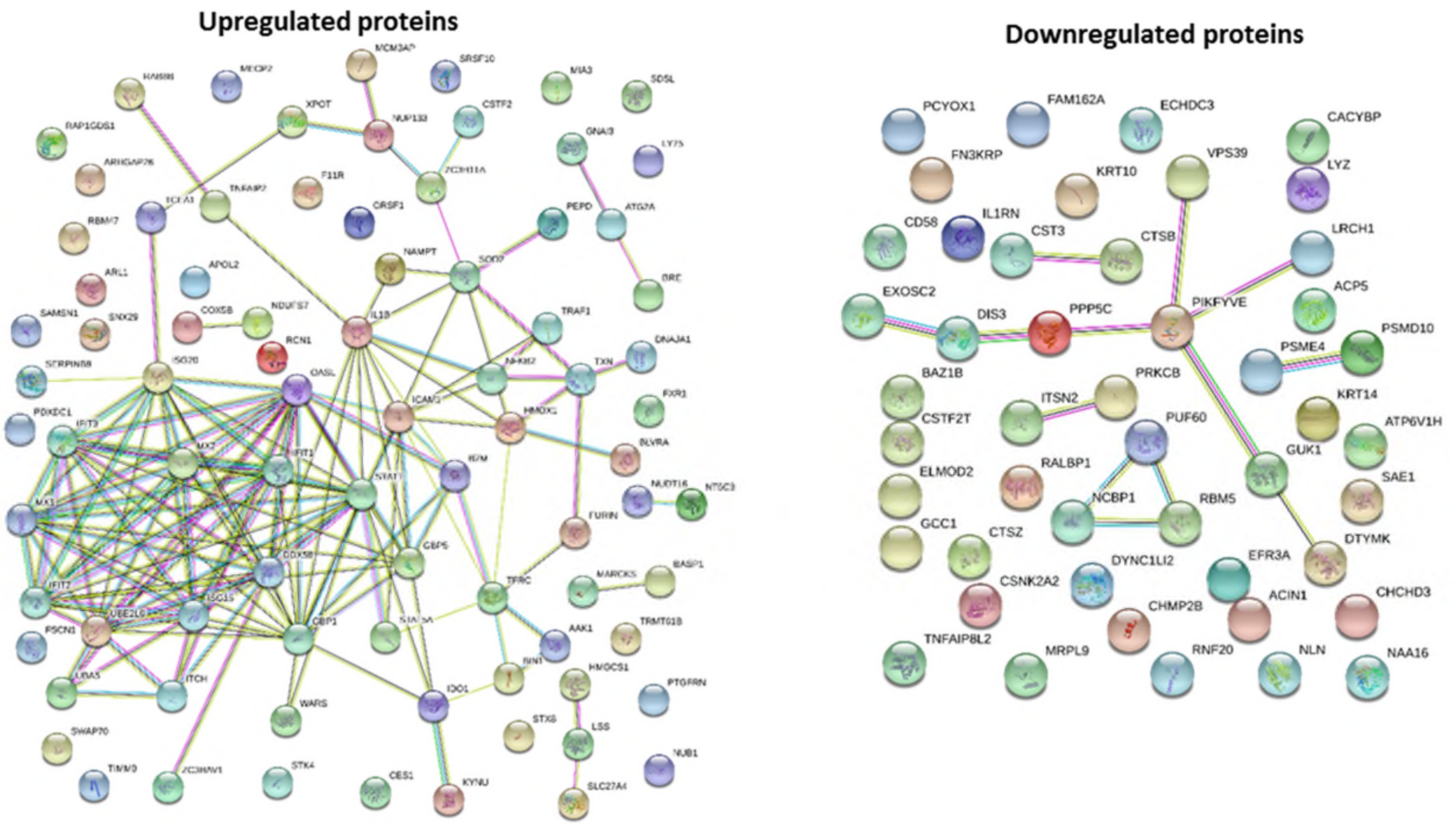

Figure S3. String analyses of dendritic cell proteins displaying significant ($p<0.05$) changes in cellular abundance (>1.5-fold) upregulation between 0 and 24 h after LPS-stimulation. Networks were drawn with seven differently coloured lines: green lines indicate neighbourhood evidence; red lines indicate the presence of gene fusion evidence; blue lines indicate co-occurrence evidence; purple lines indicate experimental evidence; yellow lines indicate text-mining evidence; light blue lines indicate database evidence; and a black line indicates co-expression evidence.

24 hours vs 6 hours

Upregulated proteins

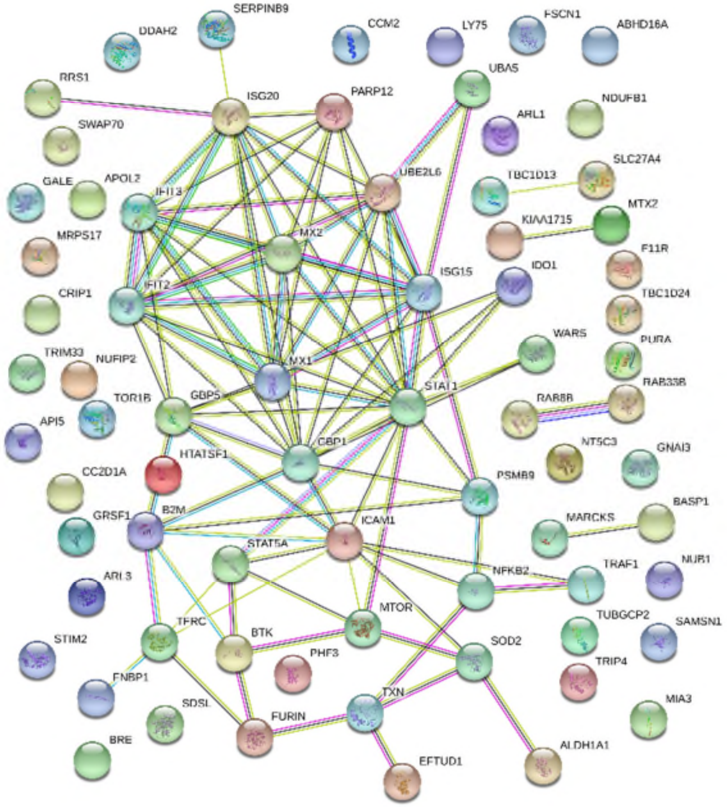

Downregulated proteins

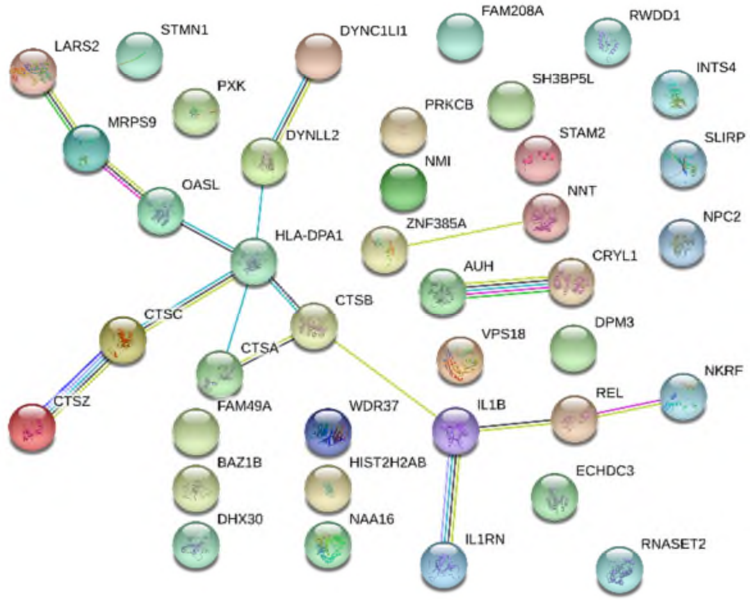

Figure S4. String analyses of dendritic cell proteins displaying significant ($p<0.05$) changes in cellular abundance (>1.5-fold) upregulation between 6 and 24 h after LPS-stimulation. Networks were drawn with seven differently coloured lines: green lines indicate neighbourhood evidence; red lines indicate the presence of gene fusion evidence; blue lines indicate co-occurrence evidence; purple lines indicate experimental evidence; yellow lines indicate text-mining evidence; light blue lines indicate database evidence; and a black line indicates co-expression evidence.

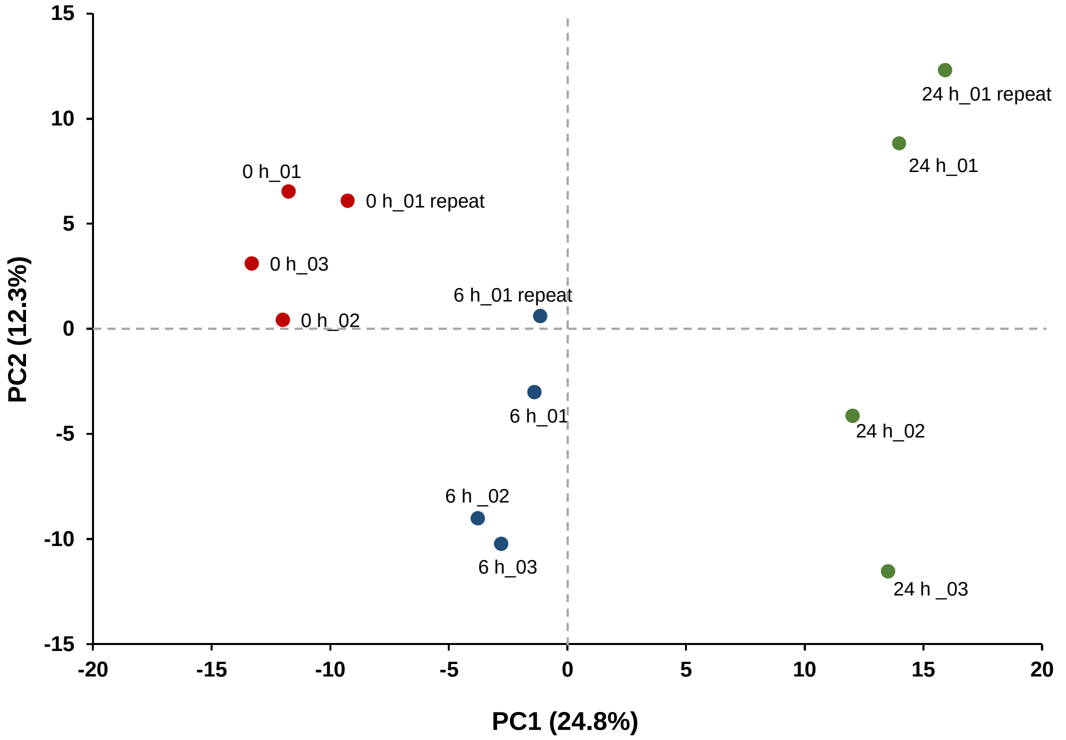

Figure S5: Principal component analysis of SWATH-MS data from technical and biological replicates of moDCs at 0 h, 6 h and 24 h after LPS treatment. The 3 biological replicates at each time-point are labelled as 01-03, respectively. The technical replicates are indicated as repeats of the “01” sample.

Table S2: Proteins significantly upregulated (p-value≤0.05) at 6 h as compared to 0 h post LPS-stimulation.

Rank	Protein Accession	Uniprot Name	Fold Change	Log_2 Fold Change	P-Value	Adjusted P-Value	Standard Error
1	P01584	IL1B	21.69	4.44	3.973E-05	4.870E-02	2.262E-01
2	Q15646	OASL	9.44	3.24	3.404E-03	3.101E-01	5.209E-01
3	Q5T2E6	CJ076	7.51	2.91	9.555E-04	2.117E-01	3.339E-01
4	P05161	ISG15	6.04	2.60	2.845E-05	4.730E-02	1.215E-01
5	P14902	I23O1	5.59	2.48	7.665E-03	4.589E-01	5.001E-01
6	Q96AZ6	ISG20	5.55	2.47	2.293E-05	4.730E-02	1.096E-01
7	O60318	GANP	5.12	2.36	2.480E-03	2.716E-01	1.176E-01
8	Q5VZ89	DEN4C	4.95	2.31	4.397E-05	4.870E-02	6.264E-02
9	Q13077	TRAF1	4.76	2.25	1.496E-02	5.625E-01	5.502E-01
10	P09913	IFIT2	4.72	2.24	1.062E-02	4.835E-01	3.915E-01
11	P20591	MX1	3.82	1.94	1.570E-03	2.429E-01	2.531E-01
12	Q14137	BOP1	3.54	1.82	3.817E-02	7.704E-01	5.983E-01
13	P29966	MARCS	3.41	1.77	1.574E-02	5.769E-01	4.397E-01
14	P15498	VAV	3.24	1.70	6.651E-03	4.257E-01	1.391E-01
15	P05362	ICAM1	3.18	1.67	1.480E-04	9.460E-02	1.187E-01
16	Q7Z2W4	ZCCHV	3.17	1.66	1.566E-04	9.460E-02	1.199E-01
17	Q7L8J4	3BP5L	3.14	1.65	1.577E-03	2.429E-01	1.490E-01
18	O14879	IFIT3	2.98	1.57	1.911E-03	2.429E-01	2.167E-01
19	Q96PP8	GBP5	2.95	1.56	1.040E-03	2.160E-01	1.832E-01
20	O95786	DDX58	2.66	1.41	4.257E-04	1.768E-01	1.312E-01
21	Q9H446	RWDD1	2.53	1.34	5.299E-03	3.947E-01	9.793E-02
22	P04179	SODM	2.43	1.28	2.653E-03	2.798E-01	1.928E-01
23	Q96RQ3	MCCA	2.42	1.28	1.498E-02	5.625E-01	3.122E-01
24	Q9Y5J7	TIM9	2.41	1.27	4.299E-02	7.736E-01	3.759E-01
25	O00148	DX39A	2.31	1.21	4.094E-03	3.534E-01	2.039E-01
26	O00499	BIN1	2.24	1.17	1.157E-02	4.955E-01	2.121E-02
27	P30519	HMOX2	2.23	1.16	8.661E-04	2.107E-01	1.297E-01
28	Q03169	TNAP2	2.23	1.15	4.423E-03	3.629E-01	1.994E-01
29	P98082	DAB2	2.19	1.13	4.761E-02	7.944E-01	2.555E-01
30	Q9H0Q0	FA49A	2.08	1.06	2.855E-02	7.013E-01	2.663E-01
31	P02746	C1QB	2.04	1.03	7.436E-03	4.493E-01	2.046E-01
32	O75886	STAM2	2.02	1.02	2.675E-02	6.913E-01	1.698E-01
33	P23381	SYWC	1.94	0.96	1.331E-03	2.429E-01	1.201E-01
34	Q96DE0	NUD16	1.93	0.95	8.592E-04	2.107E-01	1.056E-01
35	P07910	HNRPC	1.92	0.94	1.796E-03	2.429E-01	1.271E-01
36	Q8IUR7	ARMC8	1.90	0.92	5.843E-03	4.016E-01	1.309E-01
37	P04844	RPN2	1.87	0.90	4.001E-04	1.768E-01	8.240E-02
38	Q00653	NFKB2	1.84	0.88	4.160E-02	7.736E-01	2.985E-01
29	P55769	NH2L1	1.82	0.87	1.963E-03	2.429E-01	8.435E-02
40	Q8N6R0	MET13	1.78	0.83	3.862E-03	3.377E-01	1.382E-01
41	O43592	XPOT	1.70	0.76	2.095E-02	6.386E-01	2.065E-01
42	Q6P996	PDXD1	1.65	0.72	1.411E-02	5.483E-01	1.394E-01
43	Q6UW68	TM205	1.64	0.72	2.832E-03	2.852E-01	1.097E-01
44	P62805	H4	1.64	0.72	8.700E-03	4.764E-01	1.493E-01
45	P36969	GPX4	1.64	0.71	2.284E-02	6.431E-01	1.987E-01
46	O14933	UB2L6	1.64	0.71	3.115E-03	2.993E-01	1.117E-01
47	Q99627	CSN8	1.62	0.70	4.640E-02	7.944E-01	2.457E-01
48	Q16658	FSCN1	1.62	0.70	4.554E-04	1.780E-01	6.617E-02
49	Q7Z7A4	PXK	1.61	0.69	2.124E-02	6.431E-01	1.867E-01
50	P28799	GRN	1.60	0.68	1.270E-02	5.180E-01	1.265E-01
51	Q96PM9	Z385A	1.57	0.65	4.461E-02	7.835E-01	1.428E-01
52	P52306	GDS1	1.56	0.64	3.449E-02	7.414E-01	1.733E-01
53	P52815	RM12	1.55	0.63	1.814E-02	6.141E-01	8.621E-02

54	P46939	UTRO	1.54	0.62	3.899E-02	7.736E-01	1.271E-01
55	P41219	PERI	1.54	0.62	9.451E-03	4.764E-01	1.332E-01
56	Q9H7D7	WDR26	1.52	0.61	4.692E-02	7.944E-01	2.136E-01
57	P50453	SPB9	1.50	0.58	8.721E-05	6.440E-02	3.606E-02
58	P60709	ACTB	1.49	0.57	1.228E-03	2.334E-01	7.017E-02
59	P49792	RBP2	1.47	0.55	3.104E-02	7.240E-01	1.440E-01
60	P04440	DPB1	1.45	0.54	1.349E-02	5.401E-01	1.280E-01
61	Q13363	CTBP1	1.45	0.54	2.081E-02	6.373E-01	1.457E-01
62	Q13825	AUHM	1.45	0.54	1.618E-03	2.429E-01	7.057E-02
63	P10599	THIO	1.42	0.51	5.804E-03	4.016E-01	9.494E-02
64	Q8WTV0	SCRB1	1.42	0.51	2.304E-02	6.459E-01	1.421E-01
65	Q7Z6K5	ARPIN	1.42	0.51	4.298E-02	7.736E-01	1.503E-01
66	Q16719	KYNU	1.42	0.50	9.193E-04	2.107E-01	5.712E-02
67	Q01581	HMCS1	1.41	0.50	2.196E-02	6.431E-01	1.364E-01
68	P16070	CD44	1.41	0.49	5.428E-03	3.964E-01	8.981E-02
69	P62873	GBB1	1.40	0.48	4.157E-02	7.736E-01	1.625E-01
70	Q9Y608	LRRF2	1.39	0.48	8.338E-03	4.764E-01	9.860E-02
71	P10412	H14	1.39	0.48	2.862E-02	7.013E-01	1.420E-01
72	Q9BYX4	IFIH1	1.39	0.47	3.049E-02	7.240E-01	1.440E-01
73	P53007	TXTP	1.38	0.47	1.855E-02	6.186E-01	1.214E-01
74	Q5T447	HECD3	1.38	0.46	2.523E-02	6.675E-01	1.333E-01
75	Q9UK41	VPS28	1.37	0.45	1.977E-02	6.228E-01	9.871E-02
76	P08574	CY1	1.37	0.45	1.016E-02	4.764E-01	9.811E-02
77	P39656	OST48	1.36	0.45	4.184E-02	7.736E-01	1.311E-01
78	O76071	CIAO1	1.36	0.45	6.035E-03	4.093E-01	8.431E-02
79	Q3ZCM7	TBB8	1.36	0.45	2.409E-02	6.615E-01	1.259E-01
80	Q8NBQ5	DHB11	1.35	0.44	8.623E-03	4.764E-01	9.113E-02
81	P61769	B2MG	1.35	0.43	1.027E-03	2.160E-01	5.078E-02
82	P09914	IFIT1	1.35	0.43	5.002E-03	3.821E-01	7.741E-02
83	Q9Y5S1	TRPV2	1.34	0.43	2.501E-02	6.675E-01	1.217E-01
84	P23193	TCEA1	1.34	0.42	7.658E-04	2.107E-01	4.544E-02
85	P17706	PTN2	1.33	0.41	3.161E-02	7.240E-01	1.277E-01
86	Q9Y696	CLIC4	1.33	0.41	6.888E-03	4.360E-01	8.061E-02
87	Q14164	IKKE	1.33	0.41	3.194E-02	7.269E-01	1.266E-01
88	P62834	RAP1A	1.33	0.41	1.762E-03	2.429E-01	1.710E-02
89	P01903	DRA	1.32	0.40	7.594E-04	2.107E-01	4.352E-02
90	Q9UBV8	PEF1	1.31	0.39	3.322E-02	7.385E-01	1.216E-01
91	P42229	STA5A	1.30	0.38	3.606E-02	7.583E-01	1.218E-01
92	P55809	SCOT1	1.29	0.37	4.768E-02	7.944E-01	1.304E-01
93	P13674	P4HA1	1.28	0.36	3.525E-02	7.486E-01	1.149E-01
94	Q9ULA0	DNPEP	1.28	0.35	4.234E-02	7.736E-01	1.034E-01
95	Q7KZI7	MARK2	1.27	0.35	4.038E-02	7.736E-01	9.996E-02
96	P62316	SMD2	1.27	0.35	2.995E-02	7.186E-01	8.883E-02
97	O00116	ADAS	1.27	0.34	2.533E-03	2.716E-01	5.119E-02
98	P13693	TCTP	1.26	0.33	3.882E-02	7.736E-01	1.105E-01
99	P31689	DNJA1	1.26	0.33	4.346E-02	7.736E-01	1.126E-01
100	P04222	1C03	1.25	0.33	6.419E-03	4.224E-01	6.265E-02
101	Q9UIB8	SLAF5	1.25	0.32	4.231E-03	3.605E-01	5.495E-02
102	P02774	VTDB	1.25	0.32	1.402E-02	5.481E-01	7.636E-02
103	P30481	1B44	1.24	0.31	9.732E-03	4.764E-01	6.718E-02
104	Q9UBQ5	EIF3K	1.24	0.31	3.309E-02	7.385E-01	9.573E-02
105	Q9Y3A6	TMED5	1.24	0.30	3.932E-02	7.736E-01	1.011E-01
106	O43768	ENSA	1.23	0.30	4.353E-02	7.736E-01	1.023E-01
107	P49755	TMEDA	1.23	0.30	1.391E-02	5.471E-01	7.081E-02
108	P20290	BTF3	1.23	0.30	3.378E-02	7.385E-01	9.316E-02
109	Q14966	ZN638	1.23	0.29	2.391E-02	6.594E-01	8.295E-02
110	Q9H4M9	EHD1	1.22	0.29	4.831E-03	3.733E-01	5.147E-02
111	Q13057	COASY	1.22	0.28	2.495E-02	6.675E-01	8.054E-02

112	P47756	CAPZB	1.21	0.28	3.901E-02	7.736E-01	1.697E-02
113	Q15080	NCF4	1.21	0.27	1.538E-02	5.725E-01	6.686E-02
114	Q9ULZ3	ASC	1.20	0.26	4.733E-02	7.944E-01	9.256E-02
115	P63241	IF5A1	1.20	0.26	3.953E-02	7.736E-01	8.659E-02
116	P62495	ERF1	1.19	0.25	1.697E-02	6.022E-01	6.395E-02
117	Q06210	GFPT1	1.18	0.24	1.936E-02	6.186E-01	6.462E-02
118	P61158	ARP3	1.18	0.24	2.460E-02	6.675E-01	6.938E-02
119	Q9UHR5	S30BP	1.18	0.24	1.137E-02	4.939E-01	5.467E-02
120	P20292	AL5AP	1.18	0.24	4.920E-02	8.055E-01	8.547E-02
121	Q49A26	GLYR1	1.17	0.23	2.479E-03	2.716E-01	2.434E-02
122	P52597	HNRPF	1.17	0.23	3.170E-02	7.240E-01	7.001E-02
123	Q9BTT0	AN32E	1.17	0.23	2.831E-02	7.013E-01	6.708E-02
124	O15372	EIF3H	1.17	0.22	4.578E-02	7.944E-01	7.843E-02
125	Q9Y4A5	TRRAP	1.17	0.22	3.150E-02	7.240E-01	5.782E-02
126	O15143	ARC1B	1.16	0.21	3.065E-03	2.993E-01	2.376E-02
127	Q9Y2I1	NISCH	1.15	0.20	1.375E-02	5.439E-01	4.797E-02
128	O00584	RNT2	1.14	0.19	3.396E-02	7.385E-01	5.081E-02
129	Q8TF42	UBS3B	1.13	0.18	1.184E-02	5.007E-01	4.065E-02
130	P46779	RL28	1.13	0.18	4.404E-02	7.805E-01	6.114E-02
131	Q12904	AIMP1	1.13	0.18	2.234E-02	6.431E-01	4.900E-02
132	P49754	VPS41	1.12	0.16	2.077E-02	6.373E-01	3.565E-02
133	Q9NVS9	PNPO	1.11	0.15	4.773E-02	7.944E-01	5.486E-02
134	Q5TDH0	DDI2	1.11	0.15	1.779E-02	6.120E-01	3.210E-02
135	O75312	ZPR1	1.10	0.14	3.796E-02	7.704E-01	4.630E-02
136	O43426	SYNJ1	1.09	0.13	3.583E-02	7.559E-01	4.064E-02
137	Q9BQE5	APOL2	1.09	0.12	6.277E-03	4.172E-01	2.309E-02
138	Q9NT62	ATG3	1.08	0.10	3.438E-03	3.101E-01	1.692E-02
139	Q9Y606	TRUA	1.07	0.10	2.993E-02	7.186E-01	2.979E-02
140	P15311	EZRI	1.07	0.10	1.430E-02	5.494E-01	1.162E-02
141	Q6P4Q7	CNNM4	1.06	0.08	3.073E-02	7.240E-01	2.595E-02
142	Q14697	GANAB	1.05	0.07	4.698E-02	7.944E-01	2.212E-02

Table S3: Protein significantly downregulated (p-value≤0.05) at 6 h as compared to 0 h post LPS-stimulation.

Rank	Protein Accession	Uniprot name	Fold Change 6/0	Log_2 Fold Change	P-Value	Adjusted P-Value	Standard Error
1	P54725	RD23A	0.20	-2.30	2.603E-04	1.331E-01	1.888E-01
2	Q13501	SQSTM	0.22	-2.19	9.064E-03	4.764E-01	3.628E-01
3	Q9P246	STIM2	0.25	-2.01	2.342E-03	2.716E-01	2.927E-01
4	Q96BI1	S22AI	0.25	-2.00	1.419E-02	5.483E-01	4.810E-01
5	Q9Y2I7	FYV1	0.31	-1.68	1.132E-02	4.939E-01	3.777E-01
6	O75438	NDUB1	0.35	-1.53	1.163E-02	4.955E-01	1.663E-01
7	P07686	HEXB	0.37	-1.45	5.608E-04	1.983E-01	1.444E-01
8	Q9UI12	VATH	0.38	-1.40	1.018E-02	4.764E-01	1.428E-01
9	O14657	TOR1B	0.38	-1.40	4.895E-02	8.055E-01	5.016E-01
10	Q9NTM9	CUTC	0.41	-1.28	2.775E-03	2.837E-01	1.407E-01
11	Q9BTE1	DCTN5	0.41	-1.27	4.213E-02	7.736E-01	4.326E-01
12	Q14249	NUCG	0.41	-1.27	3.386E-02	7.385E-01	4.018E-01
13	Q14156	EFR3A	0.42	-1.26	2.904E-02	7.045E-01	3.206E-01
14	Q08AM6	VAC14	0.43	-1.23	4.308E-02	7.736E-01	2.641E-01
15	Q96KG9	NTKL	0.43	-1.22	4.644E-02	7.944E-01	4.267E-01
16	Q14997	PSME4	0.44	-1.17	1.331E-02	5.360E-01	2.217E-01
17	Q16644	MAPK3	0.45	-1.14	4.223E-02	7.736E-01	3.353E-01
18	Q6ZSR9	YJ005	0.46	-1.12	4.368E-03	3.629E-01	1.435E-01
19	P08571	CD14	0.48	-1.06	3.928E-02	7.736E-01	3.507E-01
20	Q9BUL8	PDC10	0.48	-1.05	1.948E-02	6.186E-01	2.769E-01
21	O75746	CMC1	0.52	-0.94	6.507E-03	4.240E-01	9.585E-03
22	Q9H665	IGFR1	0.56	-0.83	2.578E-02	6.700E-01	2.412E-01
23	P02768	ALBU	0.56	-0.83	8.579E-03	4.764E-01	1.119E-02
24	O75431	MTX2	0.56	-0.83	5.003E-02	8.057E-01	2.985E-01
25	O43719	HTSF1	0.58	-0.80	4.077E-02	7.736E-01	2.677E-01
26	Q9BXR0	TGT	0.58	-0.79	3.636E-02	7.586E-01	4.490E-02
27	P61803	DAD1	0.58	-0.77	1.606E-02	5.770E-01	1.572E-01
28	P52630	STAT2	0.59	-0.76	4.662E-02	7.944E-01	2.688E-01
29	Q9UKV3	ACINU	0.59	-0.76	4.958E-02	8.057E-01	1.763E-01
30	Q9NX63	MIC19	0.59	-0.76	2.793E-02	7.013E-01	2.237E-01
31	Q9ULP9	TBC24	0.60	-0.74	8.898E-03	4.764E-01	1.223E-01
32	Q96QR8	PURB	0.60	-0.73	2.072E-02	6.373E-01	1.978E-01
33	Q9C0E8	LNP	0.61	-0.72	2.077E-03	2.510E-01	1.019E-01
34	P21397	AOFA	0.61	-0.71	7.903E-03	4.690E-01	8.858E-03
35	P36405	ARL3	0.63	-0.67	1.136E-02	4.939E-01	1.499E-01
36	Q12846	STX4	0.63	-0.66	4.510E-02	7.868E-01	2.300E-01
37	Q08J23	NSUN2	0.64	-0.65	3.121E-02	7.240E-01	3.204E-02
38	Q7Z591	AKNA	0.64	-0.65	4.140E-02	7.736E-01	1.885E-01
29	P04181	OAT	0.64	-0.63	2.581E-02	6.700E-01	1.830E-01
40	Q9H0J9	PAR12	0.66	-0.60	4.532E-02	7.885E-01	2.091E-01
41	Q9NZT2	OGFR	0.67	-0.58	3.421E-02	7.385E-01	1.849E-01
42	P01034	CYTC	0.67	-0.58	6.930E-04	2.107E-01	6.086E-02
43	O60499	STX10	0.67	-0.58	4.195E-02	7.736E-01	1.950E-01
44	Q53GL7	PAR10	0.67	-0.57	3.821E-02	7.704E-01	1.884E-01
45	Q9HB71	CYBP	0.68	-0.56	2.524E-02	6.675E-01	1.595E-01
46	Q13094	LCP2	0.68	-0.55	1.955E-02	6.186E-01	1.200E-01
47	P38117	ETFB	0.69	-0.54	5.669E-04	1.983E-01	5.399E-02
48	Q14149	MORC3	0.70	-0.52	4.265E-02	7.736E-01	3.479E-02
49	P05023	AT1A1	0.70	-0.51	1.925E-02	6.186E-01	1.100E-01
50	O95801	TTC4	0.71	-0.50	1.315E-02	5.329E-01	1.174E-01
51	Q96I15	SCLY	0.72	-0.47	3.246E-03	3.039E-01	7.476E-02
52	Q99536	VAT1	0.72	-0.47	2.024E-02	6.326E-01	1.030E-01

53	P13861	KAP2	0.73	-0.46	2.772E-02	7.013E-01	1.364E-01
54	P36542	ATPG	0.73	-0.45	2.433E-02	6.653E-01	1.058E-01
55	Q12765	SCRN1	0.74	-0.43	4.501E-02	7.868E-01	1.479E-01
56	P68871	HBB	0.74	-0.43	3.421E-02	7.385E-01	1.348E-01
57	Q9UJY5	GGA1	0.74	-0.43	1.001E-02	4.764E-01	9.246E-02
58	O00159	MYO1C	0.75	-0.41	4.478E-03	3.629E-01	2.782E-02
59	O95352	ATG7	0.77	-0.39	4.082E-02	7.736E-01	1.118E-01
60	O00329	PK3CD	0.77	-0.38	4.969E-02	8.057E-01	2.955E-02
61	P35270	SPRE	0.77	-0.37	1.006E-02	4.764E-01	8.003E-02
62	P22570	ADRO	0.78	-0.35	2.531E-02	6.675E-01	1.014E-01
63	Q9NSY1	BMP2K	0.79	-0.34	3.813E-02	7.704E-01	1.113E-01
64	Q9H4A3	WNK1	0.79	-0.34	9.665E-03	4.764E-01	7.257E-02
65	Q9Y3I0	RTCB	0.80	-0.33	4.703E-02	7.944E-01	1.167E-01
66	P63220	RS21	0.82	-0.29	3.209E-02	7.280E-01	8.953E-02
67	Q8NFC6	BD1L1	0.82	-0.28	1.725E-02	6.034E-01	7.255E-02
68	P29401	TKT	0.83	-0.27	9.398E-03	4.764E-01	4.531E-02
69	Q9UI08	EVL	0.83	-0.27	1.904E-02	6.186E-01	7.008E-02
70	Q15208	STK38	0.84	-0.26	2.155E-02	6.431E-01	7.004E-02
71	Q9Y3D6	FIS1	0.84	-0.26	4.349E-02	7.736E-01	8.750E-02
72	Q6NVY1	HIBCH	0.85	-0.23	1.820E-02	6.141E-01	5.964E-02
73	P02671	FIBA	0.86	-0.21	2.027E-02	6.326E-01	3.078E-02
74	O43765	SGTA	0.87	-0.21	4.918E-02	8.055E-01	4.820E-02
75	Q8WUY1	THEM6	0.87	-0.21	1.060E-02	4.835E-01	4.554E-02
76	Q9HA77	SYCM	0.87	-0.20	3.779E-02	7.704E-01	5.653E-02
77	Q14683	SMC1A	0.87	-0.19	3.364E-02	7.385E-01	1.027E-02
78	Q96RU3	FNBP1	0.87	-0.19	9.881E-03	4.764E-01	3.304E-02
79	P98171	RHG04	0.88	-0.19	2.824E-02	7.013E-01	3.270E-02
80	O60664	PLIN3	0.89	-0.17	2.232E-02	6.431E-01	6.010E-03
81	P28072	PSB6	0.90	-0.15	7.127E-03	4.417E-01	3.026E-02
82	P62913	RL11	0.91	-0.14	1.886E-02	6.186E-01	3.732E-02
83	Q9H2P0	ADNP	0.91	-0.14	5.862E-03	4.016E-01	2.632E-02
84	Q03405	UPAR	0.92	-0.12	2.282E-02	6.431E-01	3.443E-02
85	O95248	MTMR5	0.92	-0.12	3.165E-02	7.240E-01	3.657E-02

Table S4: Proteins significantly upregulated (p-value≤0.05) at 24 h as compared to 0 h post LPS-stimulation.

Rank	Protein Accession	Uniprot name	Fold Change 6/0	Log2 Fold Change	P-Value	Adjusted P-Value	Standard Error
1	Q96AZ6	ISG20	24.34	4.61	6.490E-06	6.300E-03	1.488E-01
2	P14902	I23O1	19.31	4.27	9.632E-04	1.007E-01	4.912E-01
3	P05161	ISG15	17.79	4.15	1.777E-06	2.400E-03	9.697E-02
4	P20592	MX2	14.35	3.84	6.512E-03	2.948E-01	7.388E-01
5	P20591	MX1	13.97	3.80	8.759E-05	2.830E-02	2.367E-01
6	Q13077	TRAF1	12.78	3.68	2.689E-03	1.923E-01	5.546E-01
7	Q16658	FSCN1	12.49	3.64	1.191E-07	8.000E-04	4.325E-02
8	P09913	IFIT2	8.62	3.11	4.555E-03	2.468E-01	4.037E-01
9	P29966	MARCS	8.36	3.06	2.615E-03	1.910E-01	4.587E-01
10	P09958	FURIN	6.25	2.64	1.182E-02	3.684E-01	2.900E-01
11	Q9H0P0	5NT3A	5.97	2.58	1.031E-04	2.850E-02	1.671E-01
12	Q15646	OASL	5.90	2.56	6.827E-03	2.973E-01	4.988E-01
13	O14879	IFIT3	5.66	2.50	3.801E-04	6.150E-02	2.260E-01
14	Q96PP8	GBP5	5.63	2.49	1.494E-04	3.380E-02	1.776E-01
15	Q9Y2S7	PDIP2	5.36	2.42	2.518E-05	1.220E-02	1.100E-01
16	P40616	ARL1	5.35	2.42	1.230E-03	1.194E-01	2.966E-01
17	P04179	SODM	5.19	2.38	6.525E-04	8.360E-02	2.469E-01
18	P05362	ICAM1	4.86	2.28	3.399E-04	5.770E-02	2.004E-01
19	P23381	SYWC	4.72	2.24	5.299E-05	2.120E-02	1.226E-01
20	P10599	THIO	4.53	2.18	2.092E-04	3.840E-02	1.690E-01
21	O60318	GANP	4.51	2.17	1.473E-02	4.151E-01	2.666E-01
22	Q00653	NFKB2	4.36	2.12	2.175E-03	1.742E-01	3.028E-01
23	P42229	STA5A	4.34	2.12	2.419E-05	1.220E-02	9.519E-02
24	Q7Z2W4	ZCCHV	4.06	2.02	4.586E-05	1.950E-02	1.067E-01
25	P30481	1B44	3.95	1.98	1.475E-05	1.200E-02	7.870E-02
26	Q12849	GRSF1	3.85	1.94	9.363E-05	2.850E-02	1.230E-01
27	P04222	1C03	3.78	1.92	1.596E-05	1.200E-02	7.775E-02
28	Q2TAZ0	ATG2A	3.77	1.91	4.651E-03	2.468E-01	3.350E-01
29	Q9NSI8	SAMN1	3.74	1.90	1.452E-03	1.296E-01	2.436E-01
30	O14933	UB2L6	3.50	1.81	1.345E-04	3.260E-02	1.255E-01
31	P33240	CSTF2	3.39	1.76	3.433E-03	2.150E-01	2.841E-01
32	P02786	TFR1	3.37	1.75	1.483E-04	3.380E-02	1.247E-01
33	P61769	B2MG	3.30	1.72	7.175E-05	2.570E-02	1.020E-01
34	Q03169	TNAP2	3.28	1.72	7.389E-04	8.700E-02	1.841E-01
35	O95786	DDX58	3.26	1.70	1.638E-04	3.410E-02	1.242E-01
36	Q8WUM0	NU133	3.13	1.65	8.492E-03	3.258E-01	3.413E-01
37	P32455	GBP1	2.90	1.53	1.635E-03	1.406E-01	2.027E-01
38	Q9Y5J7	TIM9	2.83	1.50	7.246E-03	3.054E-01	2.976E-01
29	P42224	STAT1	2.80	1.49	1.675E-04	3.410E-02	1.091E-01
40	O75251	NDUS7	2.78	1.48	2.532E-02	5.228E-01	4.242E-01
41	Q9UH65	SWP70	2.65	1.41	2.033E-02	4.804E-01	2.036E-01
42	Q9UKY3	CES1P	2.48	1.31	2.023E-02	4.804E-01	3.509E-01
43	P80723	BASP1	2.39	1.25	4.610E-04	7.120E-02	1.192E-01
44	P50453	SPB9	2.32	1.22	2.010E-05	1.220E-02	5.217E-02
45	Q92930	RAB8B	2.31	1.21	5.881E-04	7.830E-02	1.221E-01
46	Q9UNA1	RHG26	2.27	1.18	3.237E-02	5.661E-01	3.678E-01
47	Q9BQE5	APOL2	2.21	1.14	7.232E-07	2.200E-03	2.130E-02
48	P10606	COX5B	2.18	1.13	1.182E-04	2.970E-02	4.256E-02
49	P51114	FXR1	2.13	1.09	2.290E-02	5.110E-01	3.036E-01
50	Q9BVS5	TR61B	2.10	1.07	3.672E-02	5.925E-01	3.461E-01
51	Q16719	KYNU	2.01	1.01	1.469E-03	1.296E-01	1.295E-01
52	Q9NXR7	BRE	2.00	1.00	9.698E-03	3.452E-01	1.692E-01

53	Q2M2I8	AAK1	1.99	1.00	3.830E-02	5.949E-01	3.273E-01
54	Q96J02	ITCH	1.97	0.98	2.900E-02	5.541E-01	2.941E-01
55	Q9Y624	JAM1	1.94	0.95	1.952E-02	4.685E-01	2.529E-01
56	P01584	IL1B	1.91	0.93	1.557E-02	4.264E-01	2.303E-01
57	O75152	ZC11A	1.90	0.93	4.845E-02	6.563E-01	3.305E-01
58	P09914	IFIT1	1.90	0.93	2.657E-03	1.920E-01	1.393E-01
59	O75494	SRS10	1.87	0.90	3.700E-02	5.949E-01	2.930E-01
60	P09601	HMOX1	1.85	0.89	2.304E-02	5.114E-01	1.371E-01
61	O60449	LY75	1.84	0.88	1.512E-02	4.213E-01	2.161E-01
62	O00499	BIN1	1.77	0.83	6.780E-04	8.530E-02	5.605E-02
63	Q15293	RCN1	1.76	0.81	4.037E-02	6.067E-01	2.716E-01
64	P12955	PEPD	1.72	0.78	4.790E-02	6.521E-01	2.762E-01
65	Q5JRA6	MIA3	1.71	0.78	6.986E-03	2.984E-01	1.171E-01
66	P52306	GDS1	1.71	0.78	1.572E-02	4.267E-01	9.838E-02
67	Q9Y5A7	NUB1	1.70	0.77	7.444E-03	3.065E-01	1.527E-01
68	Q13043	STK4	1.70	0.76	5.161E-03	2.541E-01	1.376E-01
69	P51608	MECP2	1.70	0.76	3.499E-02	5.784E-01	2.428E-01
70	Q9P2B2	FPRP	1.68	0.75	3.449E-03	2.150E-01	4.418E-02
71	P43490	NAMPT	1.67	0.74	2.248E-02	5.091E-01	2.046E-01
72	A0AV96	RBM47	1.67	0.74	1.450E-02	4.104E-01	1.790E-01
73	Q6P1M0	S27A4	1.66	0.73	9.046E-03	3.374E-01	1.210E-01
74	Q96GA7	SDSL	1.65	0.72	3.407E-02	5.746E-01	2.286E-01
75	P31689	DNJA1	1.64	0.72	5.427E-03	2.628E-01	1.310E-01
76	Q9UNK0	STX8	1.61	0.69	2.269E-02	5.104E-01	1.903E-01
77	Q96DE0	NUD16	1.60	0.68	2.962E-02	5.559E-01	2.058E-01
78	Q6P996	PDXD1	1.59	0.67	5.570E-03	2.665E-01	9.343E-02
79	O43592	XPOT	1.58	0.66	3.424E-02	5.746E-01	2.100E-01
80	Q01581	HMCS1	1.58	0.66	1.385E-02	4.021E-01	1.579E-01
81	P08754	GNAI3	1.58	0.66	2.891E-02	5.541E-01	1.978E-01
82	Q9GZZ9	UBA5	1.58	0.66	3.357E-02	5.715E-01	2.067E-01
83	P23193	TCEA1	1.57	0.65	3.917E-03	2.314E-01	1.081E-01
84	Q8TEQ0	SNX29	1.55	0.63	1.847E-02	4.563E-01	1.643E-01
85	P53004	BIEA	1.53	0.62	1.097E-03	1.096E-01	7.344E-02
86	P23141	EST1	1.51	0.59	1.304E-02	3.919E-01	1.120E-01
87	P48449	ERG7	1.50	0.59	4.174E-02	6.149E-01	1.987E-01
88	Q8TDW0	LRC8C	1.48	0.56	3.827E-02	5.949E-01	1.850E-01
89	O75934	SPF27	1.48	0.56	2.666E-02	5.355E-01	1.642E-01
90	Q9BYX4	IFIH1	1.48	0.56	1.776E-02	4.495E-01	1.445E-01
91	Q9UIB8	SLAF5	1.47	0.56	1.224E-03	1.194E-01	6.834E-02
92	Q9Y3Z3	SAMH1	1.47	0.55	2.700E-02	5.378E-01	9.250E-02
93	Q15181	IPYR	1.46	0.55	5.800E-03	2.755E-01	1.025E-01
94	Q13435	SF3B2	1.46	0.54	4.461E-02	6.317E-01	1.188E-01
95	Q96PP9	GBP4	1.45	0.54	4.161E-02	6.149E-01	1.826E-01
96	Q9Y3C8	UFC1	1.45	0.54	2.995E-02	5.583E-01	1.384E-01
97	P29728	OAS2	1.45	0.54	3.572E-02	5.840E-01	1.727E-01
98	Q8NBQ5	DHB11	1.45	0.53	5.067E-03	2.541E-01	9.573E-02
99	Q5JSH3	WDR44	1.44	0.53	4.117E-02	6.134E-01	3.406E-02
100	O14737	PDCD5	1.43	0.52	8.055E-03	3.180E-01	8.199E-02
101	Q99426	TBCB	1.43	0.51	1.122E-02	3.595E-01	9.147E-02
102	Q9Y5Y2	NUBP2	1.40	0.49	4.701E-02	6.451E-01	1.715E-01
103	P60709	ACTB	1.39	0.48	2.135E-03	1.742E-01	6.807E-02
104	O75746	CMC1	1.39	0.47	1.291E-02	3.897E-01	9.585E-03
105	P54922	ADPRH	1.38	0.46	4.755E-02	6.486E-01	1.430E-01
106	O95486	SC24A	1.38	0.46	4.148E-02	6.149E-01	1.565E-01
107	O75312	ZPR1	1.38	0.46	2.199E-03	1.742E-01	6.608E-02
108	Q96RU3	FNBP1	1.38	0.46	1.079E-02	3.545E-01	8.097E-02
109	P16070	CD44	1.36	0.44	8.708E-03	3.305E-01	9.178E-02
110	P40121	CAPG	1.36	0.44	1.096E-02	3.563E-01	9.784E-02

111	P19838	NFKB1	1.35	0.44	1.945E-02	4.685E-01	1.159E-01
112	Q9NVJ2	ARL8B	1.35	0.43	1.409E-02	4.037E-01	1.029E-01
113	Q7L591	DOK3	1.34	0.42	8.194E-03	3.199E-01	8.662E-02
114	O95865	DDAH2	1.33	0.42	3.568E-02	5.840E-01	1.338E-01
115	Q3ZCM7	TBB8	1.33	0.42	3.740E-02	5.949E-01	1.359E-01
116	Q96JI7	SPTCS	1.33	0.41	3.830E-02	5.949E-01	2.487E-02
117	P11836	CD20	1.33	0.41	4.910E-02	6.618E-01	1.472E-01
118	Q96QC0	PP1RA	1.33	0.41	2.544E-02	5.238E-01	1.182E-01
119	Q06210	GFPT1	1.32	0.40	1.332E-02	3.933E-01	9.346E-02
120	Q16134	ETFD	1.31	0.39	2.189E-02	5.008E-01	1.077E-01
121	Q15365	PCBP1	1.31	0.39	2.300E-03	1.775E-01	5.656E-02
122	O75131	CPNE3	1.30	0.38	7.976E-03	3.180E-01	7.775E-02
123	Q7L592	NDUF7	1.30	0.37	1.891E-02	4.626E-01	9.797E-02
124	Q06323	PSME1	1.28	0.36	3.228E-02	5.661E-01	6.621E-02
125	Q9UBF2	COPG2	1.28	0.35	2.490E-02	5.196E-01	8.466E-02
126	Q13263	TIF1B	1.27	0.35	1.528E-02	4.237E-01	6.981E-02
127	Q9Y4A5	TRRAP	1.27	0.34	1.815E-02	4.516E-01	8.852E-02
128	P30740	ILEU	1.27	0.34	1.966E-03	1.649E-01	4.733E-02
129	Q38SD2	LRRK1	1.27	0.34	3.906E-02	5.974E-01	1.125E-01
130	Q14699	RFTN1	1.26	0.33	1.046E-02	3.501E-01	7.291E-02
131	O15118	NPC1	1.26	0.33	2.280E-02	5.110E-01	9.151E-02
132	O00422	SAP18	1.26	0.33	1.513E-02	4.213E-01	8.053E-02
133	Q92688	AN32B	1.26	0.33	2.901E-02	5.541E-01	8.303E-02
134	Q9H4M9	EHD1	1.25	0.32	1.088E-04	2.850E-02	2.134E-02
135	P31949	S10AB	1.25	0.32	2.578E-02	5.275E-01	9.195E-02
136	Q96CW5	GCP3	1.24	0.31	1.313E-02	3.928E-01	5.882E-02
137	O15372	EIF3H	1.24	0.31	1.629E-02	4.357E-01	7.732E-02
138	O94855	SC24D	1.24	0.31	1.760E-02	4.495E-01	6.444E-02
139	Q6ZVF9	GRIN3	1.23	0.30	3.101E-02	5.639E-01	9.203E-02
140	P19971	TYPH	1.23	0.30	4.515E-03	2.468E-01	5.161E-02
141	Q9ULA0	DNPEP	1.23	0.30	3.640E-02	5.901E-01	9.592E-02
142	P63173	RL38	1.22	0.29	4.008E-02	6.052E-01	9.736E-02
143	P63000	RAC1	1.22	0.29	3.513E-03	2.150E-01	4.663E-02
144	P13929	ENOB	1.22	0.28	4.561E-02	6.375E-01	9.825E-02
145	Q9Y606	TRUA	1.21	0.27	9.069E-04	9.670E-02	3.101E-02
146	Q6NUK1	SCMC1	1.21	0.27	1.405E-02	4.037E-01	6.043E-03
147	O43143	DHX15	1.20	0.27	4.734E-03	2.468E-01	4.670E-02
148	P21283	VATC1	1.20	0.26	4.031E-02	6.067E-01	8.842E-02
149	Q8WX93	PALLD	1.19	0.25	4.677E-02	6.444E-01	8.958E-02
150	Q9H7D0	DOCK5	1.19	0.25	2.416E-02	5.182E-01	7.204E-02
151	Q9BUP3	HTAI2	1.19	0.25	1.698E-02	4.468E-01	6.344E-02
152	O15511	ARPC5	1.18	0.24	3.052E-02	5.620E-01	7.430E-02
153	Q14966	ZN638	1.18	0.24	1.116E-02	3.595E-01	5.371E-02
154	P48556	PSMD8	1.18	0.24	2.361E-02	5.145E-01	6.603E-02
155	P29084	T2EB	1.17	0.23	2.781E-03	1.943E-01	3.525E-02
156	Q02218	ODO1	1.17	0.23	1.400E-02	4.037E-01	2.747E-02
157	O00182	LEG9	1.17	0.22	3.776E-02	5.949E-01	7.300E-02
158	P52597	HNRPF	1.16	0.22	4.726E-02	6.472E-01	7.636E-02
159	Q13469	NFAC2	1.16	0.21	8.592E-04	9.590E-02	2.344E-02
160	P14866	HNRPL	1.15	0.21	4.256E-02	6.182E-01	4.401E-02
161	Q8TDX7	NEK7	1.15	0.21	2.139E-02	4.938E-01	5.624E-02
162	Q5T160	SYRM	1.15	0.20	3.576E-02	5.840E-01	6.375E-02
163	Q10570	CPSF1	1.15	0.20	4.143E-03	2.345E-01	2.478E-02
164	Q6UB35	C1TM	1.15	0.20	2.157E-02	4.950E-01	5.384E-02
165	P62826	RAN	1.15	0.20	6.821E-03	2.973E-01	3.819E-02
166	Q13459	MYO9B	1.14	0.19	4.447E-03	2.468E-01	3.299E-02
167	P29590	PML	1.13	0.18	8.928E-03	3.369E-01	3.734E-02
168	P53396	ACLY	1.11	0.15	1.910E-02	4.627E-01	3.880E-02

169	Q9UFN0	NPS3A	1.10	0.13	2.387E-03	1.822E-01	1.924E-02
170	Q7L014	DDX46	1.09	0.13	2.872E-02	5.541E-01	5.804E-03
171	P56385	ATP5I	1.09	0.12	2.723E-03	1.927E-01	1.871E-02
172	O43242	PSMD3	1.09	0.12	2.368E-02	5.145E-01	3.315E-02
173	O00299	CLIC1	1.07	0.10	6.895E-03	2.983E-01	1.948E-02

Table S5: Proteins significantly downregulated (p-value≤0.05) at 24 h as compared to 0 h post LPS-stimulation.

Rank	Protein Accession	Uniprot Name	Fold Change	Log 2 Fold Change	P-Value	Adjusted P-Value	Standard Error
1	P05771	KPCB	0.10	-3.25	9.940E-03	3.459E-01	3.267E-01
2	Q9UHG3	PCYOX	0.12	-3.06	2.737E-02	5.434E-01	1.317E-01
3	Q96DC8	ECHD3	0.19	-2.39	1.212E-02	3.742E-01	4.382E-01
4	Q6N069	NAA16	0.28	-1.86	9.113E-04	9.670E-02	2.106E-01
5	Q9Y2I7	FYV1	0.31	-1.69	1.068E-02	3.545E-01	3.741E-01
6	Q13868	EXOS2	0.32	-1.64	4.106E-02	6.130E-01	5.512E-01
7	Q16774	KGUA	0.34	-1.58	2.324E-02	5.121E-01	4.408E-01
8	Q14997	PSME4	0.36	-1.49	4.458E-03	2.468E-01	1.917E-01
9	Q9UIG0	BAZ1B	0.39	-1.37	1.181E-02	3.684E-01	3.114E-01
10	P19784	CSK22	0.39	-1.36	6.571E-03	2.948E-01	2.625E-01
11	P53041	PPP5	0.41	-1.28	1.091E-02	3.562E-01	1.352E-01
12	Q14156	EFR3A	0.43	-1.22	6.573E-03	2.948E-01	2.349E-01
13	Q9UI12	VATH	0.43	-1.21	2.814E-03	1.943E-01	1.331E-01
14	Q9Y2L9	LRCH1	0.44	-1.17	3.434E-02	5.746E-01	3.708E-01
15	P01034	CYTC	0.45	-1.14	1.092E-04	2.850E-02	7.493E-02
16	P52756	RBM5	0.49	-1.02	4.632E-02	6.443E-01	3.583E-01
17	Q09161	NCBP1	0.50	-0.99	4.071E-02	6.105E-01	3.312E-01
18	P19256	LFA3	0.51	-0.97	3.121E-02	5.639E-01	2.986E-01
19	Q15311	RBP1	0.51	-0.97	3.118E-02	5.639E-01	2.976E-01
20	Q9NX63	MIC19	0.52	-0.94	2.489E-02	5.196E-01	2.692E-01
21	Q5VTR2	BRE1A	0.53	-0.93	3.289E-02	5.664E-01	2.904E-01
22	P23919	KTHY	0.55	-0.85	1.655E-02	4.375E-01	2.154E-01
23	Q96CN9	GCC1	0.56	-0.83	7.283E-03	3.054E-01	1.651E-01
24	P07858	CATB	0.56	-0.83	9.949E-04	1.024E-01	9.607E-02
25	P02533	K1C14	0.57	-0.80	3.885E-02	5.972E-01	2.652E-01
26	Q9BYD2	RM09	0.57	-0.80	1.572E-02	4.267E-01	1.618E-01
27	Q9UHX1	PUF60	0.57	-0.80	2.256E-02	5.091E-01	1.221E-01
28	P61626	LYSC	0.58	-0.78	1.019E-02	3.459E-01	1.712E-01
29	Q9NZM3	ITSN2	0.58	-0.78	4.298E-02	6.199E-01	2.676E-01
30	Q9BYT8	NEUL	0.59	-0.77	4.543E-02	6.363E-01	2.315E-01
31	Q96A26	F162A	0.59	-0.76	3.508E-02	5.784E-01	2.440E-01
32	Q9HA64	KT3K	0.59	-0.75	2.244E-03	1.752E-01	7.649E-02
33	O75832	PSD10	0.60	-0.75	1.279E-03	1.207E-01	9.248E-02
34	O43237	DC1L2	0.60	-0.74	1.130E-02	3.595E-01	1.664E-01
35	P13686	PPA5	0.60	-0.73	8.538E-03	3.258E-01	1.521E-01
36	Q9HB71	CYBP	0.61	-0.71	1.266E-02	3.856E-01	1.652E-01
37	Q96JC1	VPS39	0.62	-0.69	9.840E-03	3.459E-01	1.490E-01
38	Q9UQN3	CHM2B	0.62	-0.69	6.416E-04	8.360E-02	7.124E-02
29	P13645	K1C10	0.63	-0.66	3.317E-02	5.675E-01	1.233E-01
40	Q9UKV3	ACINU	0.64	-0.65	3.376E-02	5.718E-01	1.741E-01
41	Q9UBR2	CATZ	0.64	-0.64	2.832E-03	1.943E-01	9.827E-02
42	Q9Y2L1	RRP44	0.65	-0.63	4.816E-02	6.544E-01	1.939E-01
43	Q9H0L4	CSTFT	0.65	-0.63	3.212E-02	5.661E-01	1.943E-01
44	Q6P589	TP8L2	0.65	-0.61	5.277E-04	7.790E-02	6.013E-02
45	P18510	IL1RA	0.66	-0.60	4.939E-02	6.644E-01	2.154E-01
46	Q9UBE0	SAE1	0.66	-0.60	3.300E-02	5.664E-01	1.875E-01
47	Q8IZ81	ELMD2	0.67	-0.59	1.914E-02	4.627E-01	1.546E-01
48	Q13496	MTM1	0.67	-0.58	1.008E-02	3.459E-01	1.257E-01
49	Q96ST2	IWS1	0.67	-0.57	1.260E-02	3.855E-01	1.322E-01
50	P19474	RO52	0.68	-0.57	5.430E-03	2.628E-01	1.033E-01
51	O60499	STX10	0.68	-0.55	4.180E-02	6.149E-01	1.873E-01

52	Q9NPH2	INO1	0.69	-0.53	3.782E-02	5.949E-01	1.064E-01
53	Q9Y6G9	DC1L1	0.70	-0.51	4.009E-02	6.052E-01	1.715E-01
54	O95801	TTC4	0.70	-0.51	2.145E-02	4.938E-01	1.153E-01
55	Q9Y5R8	TPPC1	0.71	-0.50	9.756E-03	3.452E-01	1.073E-01
56	O94956	SO2B1	0.71	-0.49	4.348E-02	6.231E-01	1.684E-01
57	P61313	RL15	0.71	-0.48	2.956E-02	5.559E-01	1.462E-01
58	Q9UDY2	ZO2	0.72	-0.48	1.729E-02	4.484E-01	1.220E-01
59	P04181	OAT	0.72	-0.47	3.959E-02	6.016E-01	1.570E-01
60	Q3ZCQ8	TIM50	0.72	-0.47	2.559E-02	5.251E-01	1.137E-01
61	Q9UK61	F208A	0.74	-0.44	2.658E-02	5.355E-01	1.276E-01
62	Q14318	FKBP8	0.74	-0.43	2.453E-02	5.196E-01	1.032E-01
63	Q12882	DPYD	0.75	-0.41	6.300E-03	2.931E-01	3.295E-02
64	P05387	RLA2	0.75	-0.41	3.228E-02	5.661E-01	1.274E-01
65	P20036	DPA1	0.75	-0.41	3.864E-02	5.965E-01	1.344E-01
66	P05107	ITB2	0.76	-0.40	1.900E-02	4.626E-01	8.559E-02
67	P53634	CATC	0.76	-0.39	9.090E-03	3.374E-01	8.265E-02
68	O14949	QCR8	0.76	-0.39	3.913E-02	5.974E-01	1.294E-01
69	Q8WWM7	ATX2L	0.77	-0.38	3.122E-02	5.639E-01	1.165E-01
70	Q12979	ABR	0.78	-0.36	2.470E-02	5.196E-01	1.032E-01
71	Q9Y3D6	FIS1	0.78	-0.35	3.426E-02	5.746E-01	1.117E-01
72	Q6NXE6	ARMC6	0.78	-0.35	4.286E-02	6.199E-01	1.193E-01
73	Q8IV48	ERI1	0.79	-0.35	1.811E-02	4.516E-01	9.005E-02
74	O75569	PRKRA	0.79	-0.34	1.899E-02	4.626E-01	8.992E-02
75	P28067	DMA	0.79	-0.33	4.977E-02	6.668E-01	1.191E-01
76	P38117	ETFB	0.80	-0.32	1.587E-02	4.277E-01	7.854E-02
77	Q86V48	LUZP1	0.81	-0.31	3.886E-02	5.972E-01	1.032E-01
78	P52434	RPAB3	0.81	-0.31	3.866E-03	2.314E-01	5.174E-02
79	Q9H8H3	MET7A	0.81	-0.30	2.606E-02	5.299E-01	8.791E-02
80	Q6IA86	ELP2	0.81	-0.30	2.209E-02	5.036E-01	8.258E-02
81	P30050	RL12	0.82	-0.29	2.148E-03	1.742E-01	4.120E-02
82	P02671	FIBA	0.82	-0.29	1.132E-02	3.595E-01	3.078E-02
83	Q9H299	SH3L3	0.82	-0.29	4.760E-03	2.468E-01	5.038E-02
84	P11215	ITAM	0.83	-0.27	3.606E-03	2.187E-01	4.456E-02
85	Q9NSY1	BMP2K	0.83	-0.27	4.097E-02	6.130E-01	9.175E-02
86	P08865	RSSA	0.83	-0.27	1.024E-02	3.459E-01	5.949E-02
87	P16150	LEUK	0.83	-0.27	1.823E-02	4.519E-01	7.053E-02
88	P33527	MRP1	0.84	-0.26	2.590E-02	5.284E-01	4.270E-02
89	P05556	ITB1	0.84	-0.25	2.901E-03	1.951E-01	1.154E-03
90	Q96H20	SNF8	0.84	-0.25	3.000E-02	5.583E-01	7.615E-02
91	P60866	RS20	0.84	-0.25	2.869E-02	5.541E-01	7.505E-02
92	Q96CX2	KCD12	0.85	-0.23	2.515E-02	5.215E-01	6.577E-02
93	Q96DI7	SNR40	0.85	-0.23	1.235E-02	3.796E-01	5.278E-02
94	P21281	VATB2	0.86	-0.23	3.768E-02	5.949E-01	7.354E-02
95	Q00610	CLH1	0.86	-0.22	2.744E-02	5.434E-01	3.719E-02
96	Q9C037	TRIM4	0.86	-0.21	7.918E-03	3.180E-01	4.261E-02
97	Q562R1	ACTBL	0.88	-0.19	4.909E-02	6.618E-01	6.720E-02
98	P07384	CAN1	0.88	-0.19	4.296E-02	6.199E-01	3.997E-02
99	P15880	RS2	0.88	-0.18	1.054E-03	1.068E-01	2.100E-02
100	P49755	TMEDA	0.88	-0.18	4.633E-02	6.443E-01	5.426E-02
101	Q99816	TS101	0.89	-0.17	2.921E-02	5.541E-01	5.247E-02
102	O00329	PK3CD	0.89	-0.17	1.761E-02	4.495E-01	2.273E-02
103	P13760	2B14	0.89	-0.17	9.666E-03	3.452E-01	3.623E-02
104	P78347	GTF2I	0.90	-0.16	3.484E-02	5.784E-01	4.992E-02
105	P16050	LOX15	0.91	-0.13	3.491E-02	5.784E-01	3.614E-02
106	P04040	CATA	0.93	-0.10	4.962E-02	6.661E-01	2.290E-02
107	P13489	RINI	0.94	-0.09	4.328E-02	6.226E-01	3.035E-02
108	Q05655	KPCD	0.95	-0.07	9.697E-03	3.452E-01	1.115E-03

Table S6: Proteins significantly upregulated (p-value≤0.05) at 24 h as compared to 6 h post LPS-stimulation.

Rank	Protein Accession	Uniprot Name	Fold Change	Log 2 Fold Change	P-Value	Adjusted P-Value	Standard Error
1	Q15050	RRS1	8.71	3.12	1.388E-03	1.469E-01	2.701E-01
2	P09958	FURIN	7.98	3.00	7.351E-05	2.720E-02	9.657E-02
3	Q16658	FSCN1	7.70	2.94	2.981E-06	2.800E-03	7.826E-02
4	Q9P246	STIM2	5.84	2.55	8.435E-07	2.600E-03	4.934E-02
5	Q9NVG8	TBC13	5.53	2.47	1.607E-02	4.408E-01	3.167E-01
6	P40616	ARL1	4.52	2.18	1.538E-03	1.516E-01	2.829E-01
7	Q96AZ6	ISG20	4.39	2.13	1.041E-04	3.310E-02	1.387E-01
8	Q12849	GRSF1	4.27	2.09	2.286E-05	1.270E-02	9.283E-02
9	P20591	MX1	3.65	1.87	8.969E-05	3.110E-02	1.170E-01
10	P14902	I23O1	3.45	1.79	9.321E-05	3.110E-02	1.129E-01
11	P20592	MX2	3.44	1.78	3.125E-04	6.130E-02	1.534E-01
12	Q9H0P0	5NT3A	3.38	1.76	2.742E-03	1.890E-01	2.665E-01
13	O14657	TOR1B	3.36	1.75	6.235E-03	2.907E-01	3.318E-01
14	P42229	STA5A	3.34	1.74	1.258E-04	3.360E-02	1.186E-01
15	Q15650	TRIP4	3.21	1.68	4.623E-02	6.657E-01	5.897E-01
16	P30481	1B44	3.18	1.67	2.935E-06	2.800E-03	4.424E-02
17	P10599	THIO	3.18	1.67	8.670E-04	1.230E-01	1.866E-01
18	Q9UH65	SWP70	3.08	1.62	4.604E-02	6.657E-01	3.610E-01
19	P04222	1C03	3.02	1.59	4.494E-06	3.700E-03	4.693E-02
20	P02786	TFR1	2.95	1.56	1.887E-04	4.610E-02	1.180E-01
21	P05161	ISG15	2.94	1.56	2.548E-04	5.480E-02	1.271E-01
22	Q9NSI8	SAMN1	2.77	1.47	5.433E-03	2.852E-01	2.687E-01
23	P50238	CRIP1	2.77	1.47	4.650E-02	6.666E-01	5.158E-01
24	Q13077	TRAF1	2.69	1.43	4.350E-05	2.070E-02	7.428E-02
25	O75438	NDUB1	2.67	1.42	4.962E-02	6.724E-01	3.280E-01
26	Q7Z2Z2	ETUD1	2.50	1.32	4.720E-02	6.710E-01	4.658E-01
27	O95865	DDAH2	2.49	1.32	3.573E-02	6.083E-01	4.225E-01
28	P29966	MARCS	2.45	1.29	1.488E-03	1.516E-01	1.665E-01
29	P61769	B2MG	2.45	1.29	1.889E-04	4.610E-02	9.754E-02
30	P23381	SYWC	2.43	1.28	7.947E-06	5.300E-03	4.350E-02
31	Q9BSJ2	GCP2	2.42	1.28	6.639E-03	2.951E-01	1.893E-01
32	Q9BSQ5	CCM2	2.41	1.27	2.872E-02	5.646E-01	3.801E-01
33	Q00653	NFKB2	2.36	1.24	1.818E-03	1.554E-01	1.687E-01
34	P32455	GBP1	2.36	1.24	1.936E-04	4.610E-02	9.442E-02
35	Q9NXR7	BRE	2.32	1.21	3.750E-03	2.337E-01	1.471E-01
36	Q92930	RAB8B	2.30	1.20	1.932E-03	1.609E-01	1.657E-01
37	P80723	BASP1	2.20	1.13	8.132E-04	1.198E-01	1.248E-01
38	P42224	STAT1	2.15	1.10	4.282E-03	2.464E-01	1.890E-01
29	O14933	UB2L6	2.14	1.10	8.976E-04	1.247E-01	1.239E-01
40	P04179	SODM	2.13	1.09	7.282E-03	3.079E-01	2.168E-01
41	Q06187	BTK	2.13	1.09	8.994E-03	3.392E-01	2.291E-01
42	O75431	MTX2	2.09	1.06	3.748E-02	6.233E-01	2.979E-01
43	Q14376	GALE	2.03	1.02	4.616E-02	6.657E-01	3.587E-01
44	Q9BQE5	APOL2	2.03	1.02	6.525E-07	2.600E-03	1.856E-02
45	O43719	HTSF1	2.00	1.00	4.569E-02	6.657E-01	3.478E-01
46	Q9ULP9	TBC24	1.96	0.97	2.980E-02	5.687E-01	2.484E-01
47	P28065	PSB9	1.93	0.95	8.376E-03	3.279E-01	1.520E-01
48	Q96PP8	GBP5	1.91	0.93	1.558E-03	1.516E-01	1.215E-01
49	O14879	IFIT3	1.90	0.93	6.055E-04	1.009E-01	9.444E-02
50	P00352	AL1A1	1.85	0.89	2.208E-02	5.010E-01	2.448E-01
51	P08754	GNAI3	1.83	0.87	2.063E-03	1.657E-01	1.223E-01

52	P09913	IFIT2	1.83	0.87	1.013E-03	1.308E-01	1.013E-01
53	Q00577	PURA	1.80	0.85	1.827E-02	4.702E-01	2.201E-01
54	Q7Z417	NUFP2	1.74	0.80	1.035E-02	3.561E-01	1.748E-01
55	O60449	LY75	1.73	0.79	5.645E-03	2.873E-01	1.466E-01
56	Q6P1N0	C2D1A	1.72	0.78	1.779E-02	4.669E-01	1.654E-01
57	Q9UPN9	TRI33	1.70	0.77	3.593E-02	6.083E-01	2.476E-01
58	Q9H0J9	PAR12	1.66	0.73	3.023E-02	5.724E-01	2.228E-01
59	P36405	ARL3	1.65	0.72	2.038E-03	1.657E-01	7.114E-02
60	Q9C0E8	LNP	1.64	0.71	4.299E-03	2.464E-01	1.219E-01
61	Q92576	PHF3	1.63	0.70	3.601E-02	6.083E-01	2.260E-01
62	Q6P1M0	S27A4	1.61	0.69	2.611E-02	5.283E-01	1.989E-01
63	Q9Y2R5	RT17	1.59	0.67	7.164E-03	3.079E-01	7.566E-03
64	P42345	MTOR	1.59	0.67	2.230E-02	5.010E-01	1.855E-01
65	Q9H082	RB33B	1.58	0.66	3.337E-02	5.957E-01	2.082E-01
66	Q96RU3	FNBP1	1.57	0.65	6.245E-04	1.016E-01	6.722E-02
67	Q9Y624	JAM1	1.57	0.65	1.297E-02	4.021E-01	1.523E-01
68	P50453	SPB9	1.55	0.64	1.198E-04	3.360E-02	4.280E-02
69	O95870	ABHGA	1.55	0.64	2.795E-02	5.562E-01	1.883E-01
70	Q9GZZ9	UBA5	1.54	0.62	2.726E-02	5.479E-01	1.836E-01
71	P05362	ICAM1	1.53	0.61	4.879E-02	6.724E-01	2.181E-01
72	Q5JRA6	MIA3	1.52	0.61	2.072E-02	4.969E-01	8.901E-02
73	Q9BZZ5	API5	1.52	0.61	1.182E-02	3.874E-01	1.101E-01
74	Q96GA7	SDSL	1.52	0.60	4.083E-02	6.450E-01	2.029E-01
75	Q9Y5A7	NUB1	1.51	0.59	3.702E-03	2.328E-01	9.720E-02
76	Q14790	CASP8	1.48	0.57	6.162E-03	2.907E-01	5.488E-03
77	Q13043	STK4	1.48	0.56	2.893E-04	5.840E-02	4.757E-02
78	Q03169	TNAP2	1.48	0.56	1.767E-02	4.666E-01	1.441E-01
79	O75131	CPNE3	1.47	0.56	3.942E-02	6.332E-01	1.852E-01
80	P28838	AMPL	1.45	0.53	1.395E-02	4.061E-01	1.026E-01
81	Q13555	KCC2G	1.44	0.53	2.264E-02	5.010E-01	1.469E-01
82	Q9BW92	SYTM	1.44	0.52	1.529E-03	1.516E-01	6.796E-02
83	P13861	KAP2	1.43	0.52	3.350E-03	2.190E-01	8.282E-02
84	P12277	KCRB	1.43	0.51	3.821E-03	2.351E-01	8.527E-02
85	Q5EBM0	CMPK2	1.43	0.51	9.939E-03	3.561E-01	1.109E-01
86	P36542	ATPG	1.42	0.51	4.124E-02	6.470E-01	1.470E-01
87	Q16719	KYNU	1.42	0.51	1.229E-02	3.957E-01	1.165E-01
88	Q16134	ETFD	1.42	0.50	4.591E-03	2.513E-01	8.774E-02
89	P09914	IFIT1	1.41	0.49	3.610E-02	6.083E-01	1.588E-01
90	Q7Z4V5	HDGR2	1.40	0.49	1.015E-03	1.308E-01	5.712E-02
91	Q9H0R6	GATA	1.40	0.49	2.539E-02	5.241E-01	1.404E-01
92	P40121	CAPG	1.35	0.44	1.095E-03	1.308E-01	5.182E-02
93	O75821	EIF3G	1.35	0.43	4.487E-02	6.636E-01	1.508E-01
94	Q99700	ATX2	1.35	0.43	2.385E-02	5.114E-01	1.216E-01
95	O94855	SC24D	1.35	0.43	3.844E-03	2.351E-01	5.282E-02
96	P63173	RL38	1.35	0.43	3.749E-02	6.233E-01	1.403E-01
97	Q5HYK3	COQ5	1.33	0.42	3.299E-02	5.929E-01	1.302E-01
98	P05023	AT1A1	1.33	0.41	2.387E-03	1.768E-01	6.039E-02
99	P31689	DNJA1	1.31	0.39	7.529E-03	3.137E-01	7.780E-02
100	Q8WX93	PALLD	1.30	0.38	1.714E-02	4.625E-01	7.984E-02
101	Q9UL46	PSME2	1.30	0.38	2.253E-02	5.010E-01	1.051E-01
102	Q92688	AN32B	1.30	0.38	2.728E-02	5.479E-01	1.108E-01
103	Q9ULH0	KDIS	1.30	0.37	1.555E-02	4.345E-01	9.223E-02
104	Q9NRN7	ADPPT	1.30	0.37	3.431E-02	6.017E-01	1.182E-01
105	Q9Y3I0	RTCB	1.29	0.37	4.324E-02	6.555E-01	1.255E-01
106	Q7Z2W4	ZCCHV	1.28	0.36	2.277E-02	5.010E-01	9.901E-02
107	P42858	HD	1.28	0.35	4.891E-02	6.724E-01	1.265E-01
108	Q9H223	EHD4	1.26	0.34	2.614E-02	5.283E-01	8.216E-02
109	Q15369	ELOC	1.25	0.33	4.519E-02	6.636E-01	1.137E-01

110	P54922	ADPRH	1.25	0.32	3.520E-02	6.056E-01	1.036E-01
111	Q9BRK5	CAB45	1.25	0.32	1.942E-02	4.850E-01	8.538E-02
112	O75312	ZPR1	1.25	0.32	3.126E-03	2.064E-01	5.042E-02
113	Q92918	M4K1	1.25	0.32	2.110E-02	5.010E-01	8.660E-02
114	P30740	ILEU	1.25	0.32	1.535E-03	1.516E-01	4.136E-02
115	O00159	MYO1C	1.24	0.31	2.440E-03	1.787E-01	3.233E-02
116	Q13561	DCTN2	1.23	0.30	8.410E-03	3.279E-01	4.873E-02
117	Q13636	RAB31	1.23	0.30	1.002E-02	3.561E-01	6.537E-02
118	P19838	NFKB1	1.23	0.30	2.002E-02	4.877E-01	7.999E-02
119	Q9NVJ2	ARL8B	1.23	0.30	3.646E-02	6.124E-01	9.624E-02
120	P28039	AOAH	1.23	0.30	1.345E-02	4.021E-01	6.987E-02
121	O95786	DDX58	1.23	0.29	1.257E-02	4.013E-01	6.816E-02
122	Q15628	TRADD	1.23	0.29	2.562E-02	5.254E-01	8.460E-02
123	P29084	T2EB	1.22	0.29	6.299E-03	2.914E-01	5.548E-02
124	Q15365	PCBP1	1.20	0.27	8.076E-03	3.224E-01	2.425E-02
125	B0I1T2	MYO1G	1.20	0.27	1.578E-02	4.367E-01	6.660E-02
126	Q93008	USP9X	1.18	0.24	4.497E-02	6.636E-01	8.403E-02
127	Q9UIB8	SLAF5	1.18	0.24	1.106E-02	3.723E-01	5.279E-02
128	Q08722	CD47	1.17	0.23	3.362E-02	5.971E-01	7.315E-02
129	Q96AB3	ISOC2	1.17	0.23	4.947E-02	6.724E-01	8.311E-02
130	O00161	SNP23	1.17	0.23	1.004E-02	3.561E-01	2.306E-02
131	Q7L591	DOK3	1.16	0.21	4.121E-02	6.470E-01	7.116E-02
132	P53396	ACLY	1.14	0.19	3.534E-02	6.056E-01	3.594E-02
133	P63000	RAC1	1.14	0.18	3.990E-02	6.394E-01	6.132E-02
134	O43772	MCAT	1.14	0.18	1.035E-02	3.561E-01	3.168E-02
135	P78417	GSTO1	1.13	0.18	3.847E-02	6.291E-01	5.904E-02
136	Q9Y606	TRUA	1.13	0.18	8.286E-03	3.279E-01	3.614E-02
137	Q9H2K8	TAOK3	1.13	0.18	1.342E-02	4.021E-01	4.149E-02
138	P29590	PML	1.13	0.17	8.998E-03	3.392E-01	3.626E-02
139	O75390	CISY	1.12	0.16	7.222E-03	3.079E-01	1.858E-03
140	Q9UI08	EVL	1.12	0.16	4.228E-02	6.541E-01	5.449E-02
141	P14618	KPYM	1.12	0.16	1.569E-03	1.516E-01	1.443E-02
142	Q9BUP3	HTAI2	1.10	0.14	2.888E-02	5.646E-01	4.257E-02
143	Q10570	CPSF1	1.10	0.13	2.502E-02	5.203E-01	2.131E-02
144	O00422	SAP18	1.09	0.13	2.523E-02	5.223E-01	3.752E-02
145	P52209	6PGD	1.09	0.13	2.421E-02	5.150E-01	3.036E-02
146	Q16775	GLO2	1.07	0.10	1.347E-02	4.021E-01	2.409E-02
147	Q9UBV2	SE1L1	1.07	0.10	4.564E-02	6.657E-01	2.881E-02
148	O43242	PSMD3	1.06	0.09	2.903E-02	5.659E-01	1.493E-02

Table S7: Proteins significantly downregulated (p-value≤0.05) at 24 h as compared to 6 h post LPS-stimulation.

Rank	Protein Accession	Uniprot Name	Fold Change	Log 2 Fold Change	P-Value	Adjusted P-Value	Standard Error
1	P01584	IL1B	0.09	-3.51	1.410E-06	2.600E-03	7.730E-02
2	P05771	KPCB	0.11	-3.20	1.028E-02	3.561E-01	3.267E-01
3	O15226	NKRF	0.15	-2.73	1.697E-03	1.554E-01	1.128E-01
4	Q96DC8	ECHD3	0.22	-2.18	1.803E-02	4.677E-01	4.624E-01
5	Q7L8J4	3BP5L	0.30	-1.73	1.950E-03	1.609E-01	1.682E-01
6	Q96FJ2	DYL2	0.33	-1.62	2.615E-02	5.283E-01	4.692E-01
7	Q9UIG0	BAZ1B	0.36	-1.49	1.720E-03	1.554E-01	1.990E-01
8	Q8IUE6	H2A2B	0.36	-1.46	2.004E-02	4.877E-01	3.905E-01
9	O00584	RNT2	0.42	-1.26	9.005E-03	3.392E-01	2.075E-01
10	Q6N069	NAA16	0.42	-1.25	4.866E-02	6.724E-01	4.457E-01
11	Q7Z7A4	PXK	0.44	-1.17	4.008E-03	2.407E-01	1.968E-01
12	O75886	STAM2	0.45	-1.14	4.826E-04	8.470E-02	2.511E-02
13	Q96PM9	Z385A	0.45	-1.14	4.324E-03	2.464E-01	1.458E-01
14	Q9Y2S2	CRYL1	0.46	-1.12	1.099E-03	1.308E-01	1.332E-01
15	Q9H446	RWDD1	0.47	-1.08	2.134E-02	5.010E-01	1.609E-01
16	Q9P253	VPS18	0.47	-1.08	3.102E-02	5.737E-01	1.953E-01
17	P18510	IL1RA	0.48	-1.06	4.424E-03	2.500E-01	1.829E-01
18	Q13423	NNTM	0.50	-0.99	4.532E-03	2.513E-01	6.708E-02
19	Q9GZT3	SLIRP	0.51	-0.96	4.166E-02	6.504E-01	3.253E-01
20	P10619	PPGB	0.53	-0.92	1.499E-02	4.253E-01	2.254E-01
21	P82933	RT09	0.53	-0.91	1.047E-02	3.561E-01	1.992E-01
22	P07858	CATB	0.55	-0.88	2.746E-03	1.890E-01	1.328E-01
23	Q96HW7	INT4	0.55	-0.87	4.649E-02	6.666E-01	3.040E-01
24	Q9UBR2	CATZ	0.57	-0.81	5.983E-03	2.893E-01	6.321E-02
25	P16949	STMN1	0.57	-0.81	1.560E-02	4.345E-01	1.022E-01
26	Q13287	NMI	0.59	-0.77	3.342E-02	5.957E-01	2.060E-01
27	Q15031	SYLM	0.61	-0.71	2.505E-02	5.203E-01	2.039E-01
28	Q9UK61	F208A	0.61	-0.71	3.203E-02	5.803E-01	2.203E-01
29	Q9P2X0	DPM3	0.62	-0.68	1.136E-02	3.788E-01	1.542E-01
30	Q15646	OASL	0.62	-0.68	1.888E-02	4.810E-01	1.781E-01
31	Q9H0Q0	FA49A	0.63	-0.68	4.615E-02	6.657E-01	2.062E-01
32	P61916	NPC2	0.63	-0.67	4.158E-02	6.504E-01	2.265E-01
33	P20036	DPA1	0.64	-0.65	3.574E-03	2.291E-01	1.059E-01
34	Q13825	AUHM	0.64	-0.65	3.247E-02	5.856E-01	1.714E-01
35	P53634	CATC	0.65	-0.63	1.004E-02	3.561E-01	1.083E-01
36	Q04864	REL	0.65	-0.63	3.059E-02	5.737E-01	1.625E-01
37	Q7L2E3	DHX30	0.65	-0.62	3.810E-02	6.272E-01	1.243E-01
38	Q9Y2I8	WDR37	0.65	-0.62	2.014E-02	4.883E-01	1.648E-01
29	Q9Y6G9	DC1L1	0.65	-0.61	2.225E-02	5.010E-01	1.693E-01
40	P01034	CYTC	0.68	-0.56	7.030E-04	1.088E-01	5.960E-02
41	Q8WWM7	ATX2L	0.68	-0.56	3.899E-02	6.295E-01	1.597E-01
42	Q9H4A5	GLP3L	0.68	-0.55	2.942E-02	5.687E-01	1.667E-01
43	P04440	DPB1	0.69	-0.54	2.091E-04	4.650E-02	4.223E-02
44	P01903	DRA	0.69	-0.54	1.096E-03	1.308E-01	6.412E-02
45	Q7Z4G1	COMD6	0.70	-0.52	6.805E-05	2.720E-02	3.021E-02
46	P67809	YBOX1	0.70	-0.51	1.044E-02	3.561E-01	1.122E-01
47	Q9UBP6	TRMB	0.70	-0.51	2.284E-02	5.010E-01	1.416E-01
48	Q9Y2D5	AKAP2	0.70	-0.51	2.162E-02	5.010E-01	1.386E-01
49	O95139	NDUB6	0.71	-0.50	2.396E-02	5.120E-01	1.419E-01

50	Q8WYP5	ELYS	0.71	-0.50	4.367E-02	6.555E-01	1.495E-01
51	Q15843	NEDD8	0.71	-0.49	2.986E-02	5.687E-01	1.482E-01
52	Q8IXM3	RM41	0.71	-0.49	1.359E-03	1.469E-01	6.143E-02
53	P53007	TXTP	0.72	-0.48	3.196E-02	5.803E-01	1.490E-01
54	P49755	TMEDA	0.72	-0.47	1.422E-02	4.104E-01	9.212E-02
55	P02774	VTDB	0.73	-0.46	2.811E-03	1.893E-01	7.039E-02
56	Q9GZR7	DDX24	0.73	-0.45	2.425E-02	5.150E-01	1.070E-01
57	Q6P589	TP8L2	0.74	-0.44	2.090E-02	4.995E-01	1.196E-01
58	Q9UDY2	ZO2	0.74	-0.43	4.268E-02	6.555E-01	1.279E-01
59	Q9Y5R8	TPPC1	0.74	-0.43	4.325E-02	6.555E-01	1.475E-01
60	P13686	PPA5	0.74	-0.43	6.420E-03	2.931E-01	8.232E-02
61	P06126	CD1A	0.76	-0.40	2.058E-02	4.954E-01	1.075E-01
62	Q9ULZ3	ASC	0.76	-0.40	1.721E-02	4.625E-01	1.010E-01
63	Q96CN9	GCC1	0.76	-0.39	6.212E-03	2.907E-01	7.387E-02
64	Q96GG9	DCNL1	0.77	-0.38	7.668E-03	3.156E-01	7.696E-02
65	Q9H9B4	SFXN1	0.78	-0.37	3.867E-02	6.291E-01	2.222E-02
66	P62834	RAP1A	0.78	-0.37	2.450E-02	5.154E-01	8.675E-02
67	P12081	SYHC	0.78	-0.36	9.792E-03	3.561E-01	7.800E-02
68	O43353	RIPK2	0.78	-0.36	1.117E-02	3.742E-01	8.094E-02
69	Q9H269	VPS16	0.78	-0.36	4.895E-02	6.724E-01	1.269E-01
70	P04899	GNAI2	0.78	-0.35	1.764E-02	4.666E-01	8.983E-02
71	Q07021	C1QBP	0.79	-0.35	5.154E-03	2.727E-01	6.283E-02
72	Q5Y7A7	2B1D	0.79	-0.34	2.976E-02	5.687E-01	1.040E-01
73	Q9Y265	RUVB1	0.80	-0.33	1.354E-02	4.021E-01	7.745E-02
74	Q96S66	CLCC1	0.80	-0.32	2.320E-02	5.037E-01	7.483E-02
75	P08865	RSSA	0.80	-0.32	6.624E-03	2.951E-01	6.168E-02
76	P11215	ITAM	0.81	-0.31	6.031E-03	2.893E-01	4.467E-02
77	P61960	UFM1	0.81	-0.30	6.015E-03	2.893E-01	5.707E-02
78	Q8TCS8	PNPT1	0.81	-0.30	4.368E-02	6.555E-01	2.075E-02
79	P10412	H14	0.81	-0.30	7.298E-03	3.079E-01	5.948E-02
80	P52434	RPAB3	0.82	-0.29	4.773E-02	6.724E-01	1.041E-01
81	Q07812	BAX	0.82	-0.29	1.771E-02	4.666E-01	7.542E-02
82	Q4V328	GRAP1	0.82	-0.29	1.144E-02	3.794E-01	6.589E-02
83	Q16629	SRSF7	0.82	-0.29	1.423E-02	4.104E-01	6.882E-02
84	Q9Y333	LSM2	0.83	-0.26	1.190E-02	3.874E-01	6.033E-02
85	Q9NZJ7	MTCH1	0.84	-0.26	4.687E-02	6.705E-01	9.146E-02
86	P11142	HSP7C	0.84	-0.26	5.954E-03	2.893E-01	1.987E-02
87	P13760	2B14	0.84	-0.26	1.955E-03	1.609E-01	3.534E-02
88	Q99733	NP1L4	0.84	-0.25	7.182E-04	1.088E-01	1.745E-02
89	Q5T6V5	CI064	0.84	-0.25	3.393E-02	5.986E-01	4.761E-02
90	Q14651	PLSI	0.84	-0.25	2.118E-02	5.010E-01	6.659E-02
91	P28067	DMA	0.84	-0.24	5.900E-03	2.893E-01	4.580E-02
92	P78347	GTF2I	0.85	-0.24	1.363E-02	4.021E-01	5.777E-02
93	P30050	RL12	0.85	-0.24	2.569E-02	5.254E-01	6.967E-02
94	P27338	AOFB	0.85	-0.24	3.410E-03	2.207E-01	3.882E-02
95	P47756	CAPZB	0.85	-0.24	4.501E-02	6.636E-01	1.697E-02
96	Q14185	DOCK1	0.85	-0.23	4.496E-02	6.636E-01	7.864E-02
97	P33527	MRP1	0.86	-0.23	1.316E-02	4.021E-01	4.267E-02
98	Q96CX2	KCD12	0.86	-0.22	2.189E-02	5.010E-01	6.156E-02
99	P28482	MK01	0.86	-0.21	4.032E-02	6.419E-01	7.079E-02
100	P55211	CASP9	0.87	-0.20	4.823E-02	6.724E-01	7.039E-02
101	Q9H8H3	MET7A	0.88	-0.19	4.309E-02	6.555E-01	6.533E-02
102	Q96H20	SNF8	0.88	-0.19	2.188E-02	5.010E-01	5.128E-02
103	O43747	AP1G1	0.88	-0.19	3.514E-02	6.056E-01	5.950E-02
104	P49368	TCPG	0.88	-0.18	9.427E-03	3.511E-01	3.883E-02
105	P28068	DMB	0.88	-0.18	2.611E-02	5.283E-01	5.231E-02
106	P25788	PSA3	0.89	-0.17	4.853E-02	6.724E-01	6.143E-02
107	Q5JTZ9	SYAM	0.89	-0.17	2.217E-02	5.010E-01	3.837E-02

108	Q9H299	SH3L3	0.89	-0.17	2.251E-02	5.010E-01	4.616E-02
109	Q9Y696	CLIC4	0.89	-0.16	1.696E-02	4.598E-01	3.410E-02
110	O75569	PRKRA	0.90	-0.16	1.177E-02	3.874E-01	3.600E-02
111	P13489	RINI	0.90	-0.15	2.309E-02	5.031E-01	4.312E-02
112	Q9HDC9	APMAP	0.90	-0.15	3.200E-02	5.803E-01	4.581E-02
113	Q14847	LASP1	0.91	-0.14	1.983E-02	4.877E-01	3.696E-02
114	Q9NRX4	PHP14	0.91	-0.13	1.754E-02	4.666E-01	3.415E-02
115	P62495	ERF1	0.91	-0.13	4.022E-02	6.419E-01	4.405E-02
116	P39687	AN32A	0.92	-0.12	2.959E-02	5.687E-01	3.685E-02
117	P16050	LOX15	0.92	-0.12	1.909E-02	4.810E-01	2.630E-02
118	P63010	AP2B1	0.92	-0.12	2.831E-02	5.601E-01	3.030E-02
119	P23368	MAOM	0.93	-0.11	2.026E-02	4.895E-01	2.838E-02
120	Q7L5N1	CSN6	0.93	-0.10	2.343E-02	5.072E-01	2.721E-02
121	O43598	DNPH1	0.94	-0.10	4.637E-03	2.513E-01	1.678E-02
122	P60709	ACTB	0.94	-0.09	4.954E-02	6.724E-01	3.326E-02
123	P62937	PPIA	0.95	-0.08	2.133E-02	5.010E-01	1.841E-02
124	Q9UJ70	NAGK	0.95	-0.07	4.405E-02	6.585E-01	2.492E-02
125	P46940	IQGA1	0.96	-0.06	2.830E-02	5.601E-01	2.704E-03
